# Niacin-mediated rejuvenation of macrophage/microglia enhances remyelination of the aging central nervous system

**DOI:** 10.1007/s00401-020-02129-7

**Published:** 2020-02-06

**Authors:** Khalil S. Rawji, Adam M.H. Young, Tanay Ghosh, Nathan J. Michaels, Reza Mirzaei, Janson Kappen, Kathleen L. Kolehmainen, Nima Alaeiilkhchi, Brian Lozinski, Manoj K. Mishra, Annie Pu, Weiwen Tang, Salma Zein, Deepak K. Kaushik, Michael B. Keough, Jason R. Plemel, Fiona Calvert, Andrew J. Knights, Daniel J. Gaffney, Wolfram Tetzlaff, Robin J. M. Franklin, V. Wee Yong

**Affiliations:** 1 Department of Clinical Neurosciences, Hotchkiss Brain Institute, University of Calgary, 3330 Hospital Drive, Calgary, AB T2N 4N1 Canada; 2 Wellcome-MRC Cambridge Stem Cell Institute, University of Cambridge, Cambridge, UK; 3 University of British Columbia, ICORD, Vancouver, Canada; 4 University of Alberta, Edmonton, Canada; 5 Wellcome Trust Sanger Institute, Hinxton, Cambridge, UK

**Keywords:** Remyelination, Microglia, Macrophages, Aging, Oligodendrocyte progenitor cells, Phagocytosis

## Abstract

Remyelination following CNS demyelination restores rapid signal propagation and protects axons; however, its efficiency declines with increasing age. Both intrinsic changes in the oligodendrocyte progenitor cell population and extrinsic factors in the lesion microenvironment of older subjects contribute to this decline. Microglia and monocyte-derived macrophages are critical for successful remyelination, releasing growth factors and clearing inhibitory myelin debris. Several studies have implicated delayed recruitment of macrophages/microglia into lesions as a key contributor to the decline in remyelination observed in older subjects. Here we show that the decreased expression of the scavenger receptor CD36 of aging mouse microglia and human microglia in culture underlies their reduced phagocytic activity. Overexpression of CD36 in cultured microglia rescues the deficit in phagocytosis of myelin debris. By screening for clinically approved agents that stimulate macrophages/microglia, we have found that niacin (vitamin B3) upregulates CD36 expression and enhances myelin phagocytosis by microglia in culture. This increase in myelin phagocytosis is mediated through the niacin receptor (hydroxycarboxylic acid receptor 2). Genetic fate mapping and multiphoton live imaging show that systemic treatment of 9–12-month-old demyelinated mice with therapeutically relevant doses of niacin promotes myelin debris clearance in lesions by both peripherally derived macrophages and microglia. This is accompanied by enhancement of oligodendrocyte progenitor cell numbers and by improved remyelination in the treated mice. Niacin represents a safe and translationally amenable regenerative therapy for chronic demyelinating diseases such as multiple sclerosis.

**Electronic supplementary material:**

The online version of this article (10.1007/s00401-020-02129-7) contains supplementary material, which is available to authorized users.

## Introduction

The loss of the myelin sheath has significant consequences for axonal health and conduction velocity [17, 29, 42]. Although demyelinated axons have mechanisms to compensate for the loss of the insulative myelin sheath, these axons are ultimately more prone to degeneration. Remyelination is an endogenous process that restores rapid signal propagation and protects axons, but the process is usually incomplete and becomes inefficient with advanced age in rodents [47]. Both intrinsic changes in the oligodendrocyte progenitor cell (OPC) population as well as the extrinsic lesion microenvironment are thought to underlie the decreased remyelination efficiency in older animals after demyelination, likely accelerating their rate of axonal loss [5, 12, 35, 41, 45, 46].

Innate immune cells such as resident microglia and infiltrating macrophages are critical components to successful remyelination, as they release important growth factors and clear inhibitory myelin debris [5, 23, 24, 30, 41]. Several studies have associated the reduced remyelination of older animals with a dysregulated innate immune response that manifests as delayed recruitment of macrophages/microglia into lesions [53], and their deficient motility and phagocytosis of myelin debris within lesions [5, 34, 39, 41, 43]. Heterochronic parabiosis of older demyelinated mice paired with young mice resulted in infiltration of young macrophages and enhanced remyelination [41]. These results of parabiotic young macrophages altering the inhibitory lesion microenvironment of older mice suggest that pharmacological stimulation is a practical means to rejuvenate deficient macrophages/microglia to promote remyelination in older individuals.

We sought to identify a clinically approved medication to stimulate the beneficial properties of macrophages/microglia for remyelination in subjects with increasing age. By screening a library of 1040 mainly generic medications for the capacity to activate macrophages/microglia, we discovered niacin, or vitamin B3, to enhance macrophage/microglia cytokine secretion and phagocytosis in vitro. The screening results led us to investigate whether systemic niacin therapy in the lysolecithin model of demyelination could alter lesional macrophages/microglia to foster myelin repair in 9–12-month-old (henceforth referred to as middle-aged) mice. Our collective results highlight the deficits in debris removal in lesions of middle-aged compared to young 2–3-month-old animals, and they identify a novel, clinically tolerable medication that restores critical functions of macrophages/microglia to promote aging-deficient remyelination.

## Materials and methods

### Mice

All in vivo mouse experiments were conducted with ethical approval from the Animal Care Committee at the University of Calgary under regulations stated by the Canadian Council of Animal Care. Female C57Bl/6 mice from Charles River were used for lysolecithin demyelination at 2–3 months or 9–12 months of age. For the multiphoton live imaging experiments, heterozygous CX3CR1^GFP/+^ mice [18] were crossed with Thy1^YFP+^ mice [11] to produce double transgenic mice enabling the visualization of both macrophages/microglia and axons [39]. To determine whether niacin was signaling through the niacin receptor on macrophages, Hcar2^*−/−*^ mice [49] were used. To discriminate between microglia and bone marrow-derived monocytes, male and female CX3CR1^CreER^:Rosa26^Tdt^ (also referred to as Ai9) mice were employed [36].

## Lysolecithin demyelination

All surgeries were started in the morning and mice were anesthetized with an intraperitoneal injection of ketamine (100 mg/kg) and xylazine (10 mg/kg). Buprenorphine (0.05 mg/kg) was injected subcutaneously immediately prior to surgery and 12–16 h post-surgery as an analgesic. The tissue between T3 and T4 was removed with spring scissors, exposing the dorsal spinal cord, after which the dura was cleared with a 30-gauge metal needle. For all histology experiments and electron microscopy experiments, focal demyelination was produced by injection of the lipid-disrupting detergent lysolecithin (L1381, Sigma, lysophosphatidylcholine, LPC) into the ventrolateral funiculus as described previously [20]. For multiphoton live imaging experiments, LPC was injected into the dorsal column as performed before [39]. For experiments in which demyelinated mice were treated with niacin, mice were administered with either saline vehicle or niacin (100 mg/kg) intraperitoneally once a day commencing 1 day after surgery.

## Ex vivo multiphoton live imaging

Ex vivo multiphoton live imaging was performed as described previously [39] using a Nikon A1R multiphoton microscope. A 25X water-immersion apochromatic lens with a 1.1 NA was used along with a spectral detector in which emission spectra ranging from 490 to 650 nm was collected at 10 nm intervals. Images were acquired as Z-stacks with an optical thickness of ≈ 1.5 μm per slice with *x*–*y* dimensions of 200 μm by 200 μm centered over the lesion. Z-stacks were approximately 30 μm in depth and were captured over the same location every 15 min over 2–3 h. After acquisition, images were spectrally unmixed using NIS-Elements. To obtain four-dimensional morphological and dynamic data of CX3CR1^GFP/+^ cells, unmixed time series were imported into Imaris for three-dimensional surface reconstruction. ImarisTrack was used to obtain features of motility for CX3CR1^GFP/+^ cells and ImarisColoc was used to quantify the percentage of Nile Red phagocytosis.

## Histology and immunofluorescence

Mice were anesthetized and then transcardially perfused with 20 mL of cold phosphate-buffered saline (PBS) and cold 4% phosphate-buffered paraformaldehyde (PFA). After dissecting the spinal cord, a 1 cm segment encompassing the T3–T4 thoracic spinal cord was isolated. This segment was subsequently post-fixed in 4% PFA for 24 h at 4 °C, after which the samples were cryoprotected in 30% sucrose for 3 nights at 4 °C. These samples were then frozen in cryomolds with optimal cutting temperature (OCT; 25608-930, VWR) in a chamber containing 2-methylbutane (M32631, Sigma) and dry ice. Coronal sections of 20 μm thickness were stained with eriochrome cyanine R (32752, Sigma) and 1% neutral red (N-129-25, Fisher Scientific) as previously described [21] to identify the lesion epicenter.

For immunofluorescence staining, frozen slides containing sections immediately adjacent to the lesion epicenters were thawed, and sections were delipidated in graded ethanol solutions when staining for MBP. Slides after blocking were incubated overnight with primary antibodies: rabbit polyclonal anti-Olig2 antibody (EMD Millipore, AB9610; 1:200) and goat polyclonal anti-PDGFRα antibody (R&D Systems, AF1062; 1:100) for OPCs; rat monoclonal anti-MBP antibody (Abcam, ab7349; 1:100) or rabbit polyclonal anti-MBP antibody (Abcam, ab40390; 1:1000) for myelin; rat polyclonal anti-CD45 antibody (BD Pharmingen, 550539; 1:100) and rabbit polyclonal anti-Iba1 antibody (Wako, 019-19741; 1:500) for macrophages/microglia; rabbit polyclonal anti-CD36 antibody (Novus Biologicals, NB400-144; 1:100); and rabbit polyclonal anti-IL-1β antibody (Rockland, 210-401-319; 1:50). The corresponding fluorophore-conjugated secondary antibodies (1:400) were then added with nuclear yellow (1:1000). Slides were coverslipped and fluorescent images were captured on a Nikon C1si spectral confocal microscope; brightness and contrast was adjusted using ImageJ.

To histologically examine phagocytosed lipids, tissue sections were stained with Oil Red O as previously described [24, 41]. ImageJ was used to threshold the Oil Red O images, allowing the degree of punctate Oil Red O to be analyzed.

## Flow cytometry

We conducted flow cytometry to investigate the inflammatory state of circulating blood monocytes as previously described [2]. The primary antibodies used were CD45-PerCP (BD Pharmingen; Clone 30-F11), CD11b-FITC (BD Pharmingen; Clone M1/70), Ly6G-APC-Cy7 (BD Pharmingen; Clone 1A8), and Ly6C-V450 (BD Horizon; Clone AL-21). Data were acquired on a BD LSRII flow cytometer. Unstained samples, appropriate isotype controls, as well as single-stain controls were used for compensation and gating. Data were analyzed using FlowJo software.

## Transmission electron microscopy

To assess remyelination ultrastructurally, mice were anesthetized and transcardially perfused with 20 mL of ice-cold PBS followed by 20 mL of 2.5% glutaraldehyde in 4% phosphate-buffered paraformaldehyde. The spinal cord was carefully dissected and a 3 mm segment surrounding the lesion was isolated. The lesion segments were then post-fixed overnight in the same fixative at 4 °C. The lesion segments were rinsed three times with 0.1 M cacodylate. After rinsing, the segments were fixed with 1% osmium tetroxide in 0.1 M cacodylate for 1 h at room temperature. The segments were then dehydrated in graded ethanols and embedded in Epon resin for sectioning as semi-thins on a Leica EM UC6 ultramicrotome. The semi-thins were stained with 1% toluidine blue and 2% borax to determine whether lesions are present. Once lesions were identified, segments were further processed as 70 nm ultra-thin sections and then contrasted with lead citrate for examination under a Hitachi 7650 transmission electron microscope. Images were captured at 1000× magnification, ensuring that the entire lesion was captured. Images were blinded and then randomly selected for analysis. ImageTrak was used to measure the *g* ratio (diameter of the axon divided by the diameter of the axon and myelin sheath) of individual axons within the lesion core until 300 axons per lesion were measured.

## Laser capture microdissection and RNA sequencing

Young (6 weeks old) and middle-aged (8 months old) mice were perfused with RNase-free PBS and spinal cords from naïve and injured (72 h) mice were isolated, placed in optimum cutting temperature compound (Tissue-Tek), and snap frozen in a mixture of dry ice and isopentane. 20 µm cryosections were collected on nuclease free PEN-membrane slides (Zeiss), fixed in 70% ethanol (vol/vol), stained with 1% cresyl violet (wt/vol), and dehydrated with 70% and 100% ethanol. Laser capture microdissection was performed on the PALM MicroBeam. Total RNA was isolated using RNeasy Mini Kit (Qiagen) from dissected tissue. Qubit RNA HS assay kit (Thermo Fisher) was then used to determine RNA concentrations and Agilent 2200 TapeStation to assess RNA quality. RNA samples with RIN > 8 were used for sequencing.

RNA was amplified at the library building step using the REPLI-g protocol and sequencing was performed on an Illumina NextSeq500 platform. FastQC (version 0.11.4) was used to determine the quality of sequencing reads before being mapped to the genome using TopHat (version 2.0.10) with bowtie (version 1.1.2) as an internal aligner. Raw read counts were acquired with featureCounts and read into R (version 3.3.2). Pairwise comparisons were conducted using the DESeq2 package from Bioconductor. The data reported in this paper have been deposited in the Gene Expression Omnibus, https://www.ncbi.nlm.nih.gov/geo/query/acc.cgi (accession no. GSE 129000).

## Isolation of mouse bone marrow-derived macrophages, microglia and OPCs

Mouse bone marrow-derived macrophages (BMDM) were isolated as previously described [10]. For microglia, brain tissue was diced into small pieces and incubated at 37 °C for 30 min in a dissociation solution, consisting of 34 U/mL papain and 20 μg/mL DNAse in HALF (Hibernate-A equivalent, made in house). After this, tissue was triturated with a fire-polished glass pipette, passed through a 70 μm cell strainer (Millipore) and centrifuged for 20 min at 800 g in 22.5% Percoll. The pellet, containing single cells, was labeled with magnetic bead-conjugated antibodies for CD11b and CD11b+ microglia were eluted by MACS according to the manufacturer’s instructions. Microglia were cultured at 10^4^ cells per well of a poly-d-lysine-coated 96-well microplate, in DMEM/F12 supplemented with 10% fetal bovine serum (FBS), 2% B27, 500 μM *N*-acetylcysteine and 1% penicillin–streptomycin. After 48 h, medium was changed to macrophage serum-free medium (Thermo Fisher Scientific). Finally, OPCs were isolated from CD-1 pups aged P0–P2 as previously described [20].

## Biopsy of human brain tissue

Human brain tissue was obtained with informed consent under protocol 16/LO/2168 approved by the NHS Health Research Authority. Adult brain tissue biopsies were taken from the site of neurosurgery resection for the original clinical indication. Tissue was transferred to hibernate-A low fluorescence (HALF; in house made) supplemented with 1 × SOS, 2% GlutaMAX (Life Technologies), 1% P/S (Sigma), 0.1% BSA, insulin (4 μg/mL), pyruvate (220 μg/mL) and DNase 1 Type IV (40 μg/mL) on ice and transported to a CL 2 laboratory.

## Myelin isolation

Whole brains were removed from three young adult C57Bl/6 mice and myelin was isolated using a discontinuous sucrose gradient. Brains were homogenized in 20 mL 0.32 M sucrose and 10 mL of homogenate was layered between 18.5 mL 0.85 M sucrose and 8.5 mL 0.32 M sucrose and ultracentrifuged at 75,000*g* for 20 min with no brake. The interface was collected, homogenized in 35 mL distilled water and then ultracentrifuged at 75,000*g* for a further 15 min. Supernatant was aspirated, the pellet re-suspended in 35 mL distilled water, then ultracentrifuged at 12,000*g* for 10 min. The pellet was re-suspended in 10 mL 0.32 M sucrose, layered over 20 mL 0.85 M sucrose in a clean tube and then ultracentrifuged at 75,000*g* for 30 min. Finally, the interface was collected and centrifuged at 16,000*g* for 15 min to concentrate the myelin, which was stored at − 80 °C.

## Microglia stimulation assays

Cells were stimulated in macrophage serum-free medium (Thermo Fisher Scientific) with 2% B27, 500 μM *N*-acetylcysteine, 1% penicillin–streptomycin and 10 ng/mL macrophage colony-stimulating factor (M-CSF). Latex beads or myelin were used at concentrations of 0.1 μL/mL and 10 μg/mL, respectively. Sulfo-*N*-succinimidyl oleate (SSO) was used to inhibit CD36 activity at a range of concentrations up to 200 μg/mL.

## Microglia RNA extraction, quality assessment, and RNA sequencing

Total RNA from primary microglia was extracted with the Qiagen AllPrep DNA/RNA micro kit (80284). The integrity and quantity of the total RNA were assessed using an Agilent Technologies Bioanalyser RNA Pico kit (5067-1513). Between 0.3 and 10 ng of total RNA was used as input for the RNA-seq library preparation.

Genes for which the count value was equal to zero in all samples were filtered out and 41,677 genes remained for statistical analyses. Analysis of deviance (ANODEV) was performed by employing DEseq2 and a generalized linear model (GLM) design (= ag + ac + ag:ac) where we consider the effect of aging (ag), microglia activation (ac), and the interaction of these two factors (ag:ac). The likelihood ratio test (LRT) *p* values (for the effect of aging and the effect of microglia activation) were determined and adjusted to correct for multiple testing using FDR (Benjamini–Hochberg). Expression levels of genes for which FDR < 0.05 (for both the effect of aging and the effect of microglia activation) were considered differentially expressed and 1714 genes were identified. They are collectively named DAA (gene set *d*ifferentially expressed as an effect of microglia *a*ctivation and *a*ging). For specific contrast analysis between two groups, Wald test was performed and *p* values were adjusted using FDR.

## Microglia mRNA pulldown, reverse transcription, and template switching

RNA samples were diluted to 25 µL with 10 mM Tris–HCl (pH 8.0) before adding 25 µL of 2 × lysis binding buffer. 20 µL of oligo dT beads were placed into each well of a 96-well plate and placed on the magnet. Supernatant was discarded and washed once with 100 µL lysis/binding buffer. The pelleted bead was re-suspended in 50 µL RNA and incubated at room temperature for 15 min. Samples were placed on the magnet with supernatant discarded, and eluted in 50 µL. Samples were washed again and eluted in 9.5 µL elution buffer by incubating at 75 °C for 2 min. Samples were immediately transferred to the magnet and 7 µL were transferred to a new plate and kept on ice. Samples were then incubated at 72 °C for 3 min and rapidly chilled on ice. Thereafter, samples were added to 5 µL 5 × SmartScribe FS Buffer (Takara Clontech 072915), 0.63 µL SUPERase Inhibitor (Thermo Fisher AM2696), 1.25 µL 0.1 M dithiothreitol, 5 µL 5 M betaine, 0.15 µL 1 M MgCl_2_, 0.38 µL 100 µM TSO, and 1.25 µL SMARTScribe reverse transcriptase (Takara Clontech 639538). Samples were then incubated for ten cycles before 25 µL NFW (total 50 µL) was added to perform 0.8:1 Ampure XP clean-up on Zephyr. Samples were then added to 13 µL of PCR master mix comprising 12.5 µL 2 × KAPA HiFi HS (KapaBiosystems KK2601) and 0.5 µL 10 µM ISPCR primer. The samples were run for 11 cycles with an annealing temperature of 67 °C for 15 s. Samples were added to 25 µL NFW (total 50 µL) for clean up with 0.8:1 Ampure XP, eluting in 20 µL 10 mM Tris–HCl (pH 8.0). Samples were read on a BMG Pherastar.

## Nextera Library preparation and PCR

Four nanograms of cDNA was diluted into a total volume of 9.5 µL with 10 mM Tris–HCl (pH 8.0), on ice, prior to tagmentation. 5.5 µL of tagmentation mastermix (Illumina FC-121-1030) was added and briefly vortexed for 5 s. Samples were incubated at 55 °C for 5 min, and then cooled to 15 °C. To this, 2.5 µL of Tn5 release buffer was added and incubated at room temperature for 10 min. 35 µL of 10 mM Tris–HCl (pH 8.0) was added and 1.8:1 Ampure XP clean-up was performed on Pre-PCR Zephyr. Samples were eluted in 10 µL of Tris–HCl (pH 8.0). 7 µL of tagmented cDNA (allows for dead volume) was removed for PCR. The primers were Oligo-dT_30_ VN (with RNase-free HPLC purification):

5′ AAGCAGTGGTATCAACGCAGAGTACTTTTTTTTTTTTTTTTTTTTTTTTTTTTTTVN 3′

Template-switching LNA oligo (TSO): 5′ AAGCAGTGGTATCAACGCAGAGTACATrGrG+G 3′.

ISPCR primer: 5′ AAGCAGTGGTATCAACGCAGAGT 3′.

PC1 primer: 5′ AATGATACGGCGACCACCGAGATCTACAC 3′.

PC2 primer: 5′ CAAGCAGAAGACGGCATACGAGAT 3′.

## In vitro transcription

The artificial DNA templates used in the transcription reactions were generated by performing PCR using tailed primers that introduce a bacteriophage T7 promoter sequence at one end of the amplified DNA using the primers identified below. The DNA templates containing the CD36 gene were derived from microglia lysate extracted as described above. 200 µL PCR reactions were prepared to obtain sufficient levels of product. 100 ng of T7-CD36 DNA was transfected with 0.2 µL of Lipofectamine 2000 (Thermo Fisher 11668030) for 6 h after which cells were washed with 37 °C media. Media was replaced and cells were kept for 5 days before stimulation with media changes at 24 and 36 h.

CD36 T7P forward primer: 5′ TAATACGACTCACTATAGCCACCATGGGCTGTGATCGGAACTGTG 3′.

CD36 reverse primer: 5′ TTATTTTCCATTCTTGGATTTGCAAGCAC 3′.

## Macrophage phagocytosis assay and cytokine/chemokine assay

Mouse bone marrow-derived macrophages (BMDM) were plated in a 96-well black-bottom plate at a density of 30,000 cells per well in complete BMDM medium. Cells were incubated for 24 h at 37 °C with 5% CO_2_, after which the medium was changed to DMEM with 1% FBS. After 1 h incubation, niacin (100 μM) was added to the cells, after which they were incubated for 12 h at 37 °C with 5% CO_2_. The medium was then removed and pHrodo *S.aureus* BioParticles (pHrodo; Molecular Probes Invitrogen) was added at 100 μg/mL in 1× live cell imaging solution. To label live cells and nuclei, respectively, calcein AM (10 μM) and NucBlue Live ReadyProbes Reagent (two drops per mL; Thermo Fisher) were also added to the cells. The cells were then imaged on an ImageXpress Micro Cellular Imaging and Analysis System (Molecular Devices) over an hour. The percentage of calcein AM-positive and NucBlue-positive cells that were positive for pHrodo was quantified using MetaXpress software. Cytokine secretion was assessed using a tumor necrosis factor-α ELISA following the manufacturer's instructions (Life Technologies).  Multiple cytokines and chemokines were examined using a multiplex analysis (Eve Technologies).  For cytokine experiments, niacin (100 μM) was added to cells followed by the addition of lipopolysaccharide (100 ng/mL) one hour later. Cells were then incubated for 24 hours after which conditioned medium was collected for analysis.

## Experimental design and statistical analyses

For in vivo experiments, mice were randomly allocated to either the vehicle or niacin group. All microscopy analyses were performed blinded by an individual in the laboratory not involved in this particular study. Mice were housed in cages containing three to five animals in a specific pathogen-free facility. Mice were housed at room temperature with a standard 12 h light/dark cycle with ad libitum access to water and standard diet. Mice were assessed once a day prior to the experiment, twice a day for the first 7 days following surgery, and daily thereafter. Sample size was determined by previous studies using the lysolecithin model with the corresponding outcome measure. All animal experiments had between three and nine mice per group. Saline vehicle or niacin (100 mg/kg IP) dissolved in saline was administered once a day at the same time every day for the duration of the experiment. No adverse events were observed following niacin treatment.

For experiments in which two groups were compared, a one- or two-tailed *t* test was performed for parametric data; for non-parametric data such as rank order analysis, the Mann–Whitney *U* test was used. For experiments in which there were more than two groups with one independent variable, a one-way ANOVA was conducted with either a Bonferroni post hoc test or a Dunnett’s post hoc test. For experiments in which there were more than two groups with two independent variables, a two-way ANOVA was utilized with a Bonferroni post hoc test.

## Results

**Fig. 1:**
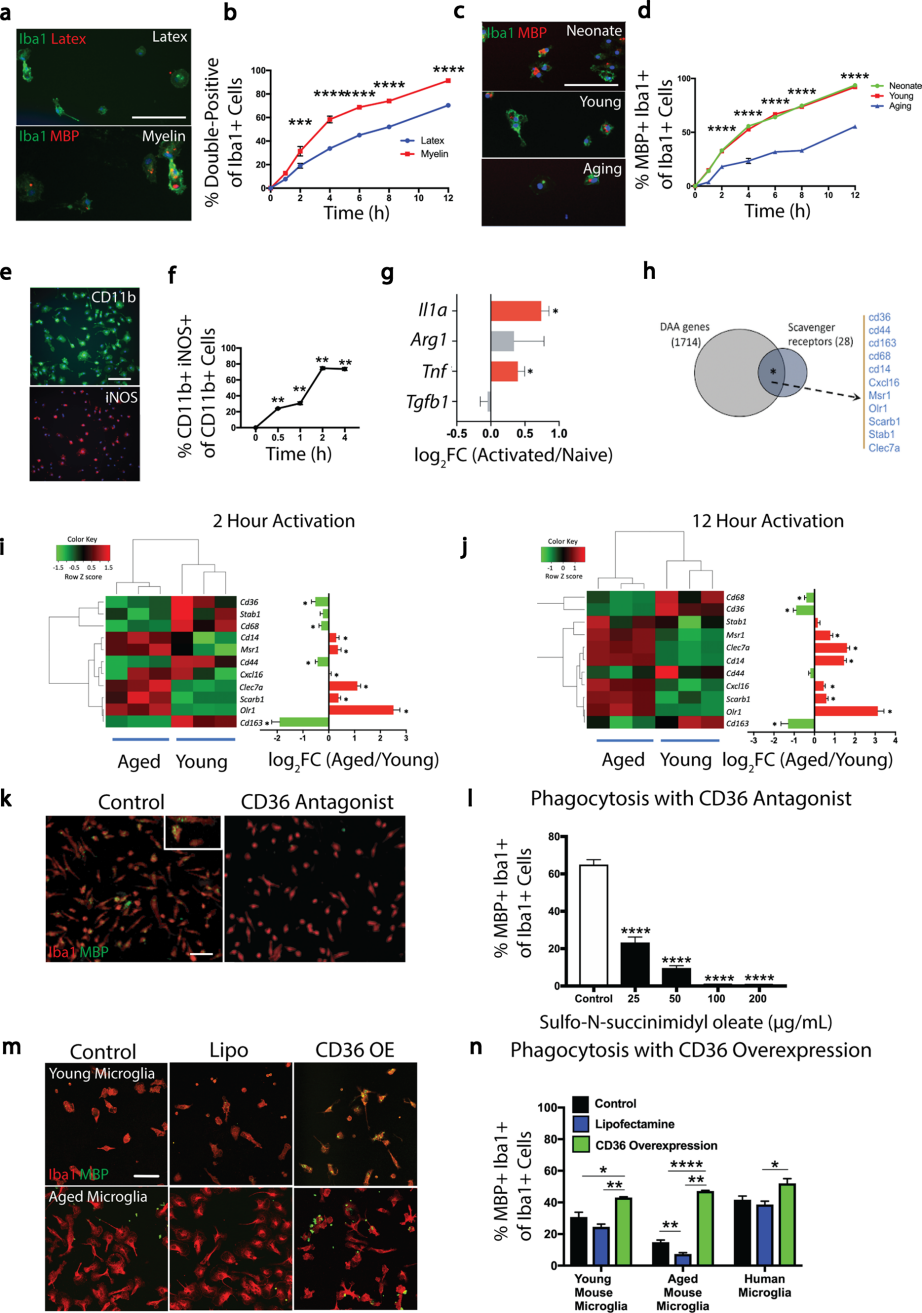
Myelin debris activates microglia and myelin phagocytosis requires the scavenger receptor CD36. **a** Representative images of mouse microglia exposed to myelin debris and fluorescently conjugated latex beads (red) and stained for Iba1 (green) and MBP (red). **b** A greater percentage of microglia phagocytose myelin debris compared to latex beads over time. **c** Representative images of neonate, young adult, and aged microglia exposed to myelin debris and stained for Iba1 (green) and MBP (red). **d** Neonate and young microglia phagocytose myelin debris with a significantly greater efficiency than aged microglia. **e** Representative images of mouse microglia exposed to myelin debris and stained for CD11b (green) and iNOS (red). **f** Myelin debris stimulates the expression of iNOS in CD11b+ microglia. **g** Log_2_ fold change of RNA expression of pro- and anti-inflammatory genes from microglia activated with myelin debris for 2 h compared to naïve controls. **h** Venn diagram representation of the association of scavenger receptors in the set of differentially expressed genes (DAA) whose expression in microglia was regulated upon activation and that is affected due to aging. **i**, **j** Heat maps of expression levels of scavenger receptors (in DAA) in microglia isolated from young and aging mice and activated for 2 (**i**) and 12 h (**j**). Euclidean distance matrix was used for hierarchical clustering. Difference in expression of scavenger receptors in microglia from aged mice was compared to microglia from young mice and the estimated log_2_ fold change was plotted. **k** Representative images of mouse microglia exposed to myelin debris and treated with a CD36 receptor antagonist (sulfo-*N*-succinimidyl oleate, SSO) and stained for Iba1 (red) and MBP (green). **l** SSO reduces microglia phagocytosis of myelin debris. **m** Representative images of young and aged microglia exposed to myelin debris and treated with control, lipofectamine (Lipo), and overexpression of CD36 (CD36 OE) and stained for Iba1 (red) and MBP (green). **n** Overexpression of CD36 significantly enhances myelin phagocytosis by young and aged mouse microglia as well as human microglia. Values are represented as mean with the standard error of the mean. Results were analyzed with a two-way repeated-measures ANOVA with a Bonferroni’s post hoc test (**b**, **d**), a one-way repeated-measures ANOVA with a Dunnett’s post hoc test (**f**, **l**), a Wald test and Benjamin–Hochberg *p* value adjustment (**g**, **i**, **j**), a two-sided Chi-square test with Yates’ correction (**h**), and a one-way ANOVA with a Bonferroni’s post hoc test (**n** each cell type analyzed individually). Values are indicative of triplicate cultures. For panels **a**, **c**, **e**, **k**, **m**, scale bars equal 50 μm. For panels **b**, **d**, **f**, **l**, **n**, *p < 0.05; **p < 0.01; ***p < 0.001; ****p < 0.0001. For panels **g**, **i**, **j**, *p < 0.05

Primary microglia were isolated from young adult (6–8 weeks) mice to assess rates of engulfment of myelin debris and latex beads (Fig. 1a). After 4 h of exposure, there was more uptake of myelin compared to latex beads, and this progressively increased with time (Fig. 1b). As phagocytosis declines with age in macrophages [34, 39], we sought to corroborate this finding in microglia from aged (> 15 month) mice. We observed a significant reduction in the uptake of myelin by microglia isolated from aged adult mice compared to microglia isolated from neonate or young adult mice (Fig. 1c, d).

The results of preferential phagocytosis of myelin debris over inert beads suggest the involvement of specific receptor(s). To address this, we first determined the time lag between microglia activation and phagocytosis. Microglia were exposed to myelin debris and staining for intracellular iNOS demonstrate that 80% of cells were stimulated after 2 h (Fig. 1e, f). We thus chose the 2 h time point for RNA sequencing and assessed the expression of selected signature genes associated with pro-inflammatory (*Il1α*, *Tnf*) or anti-inflammatory (*Arg1*, *Tgfb1*) microglia activity. Myelin debris upregulated the pro-inflammatory *Il1α* and *Tnf* but not the anti-inflammatory genes (Fig. 1g). Overall, 1714 genes were differentially expressed with age and activation state in myelin-stimulated young and aged microglia (Fig. 1h).

To determine the receptor(s) mediating phagocytosis of myelin debris, we examined the RNA sequencing dataset for genes differentially expressed with age and activation state (DAA). When comparing a scavenger receptor gene set to the DAA genes, we observed an overlap of 39.28% (11 out of 28 scavenger receptor genes; Fig. 1h). At 2 h and another 12 h time point, we found significant alterations in the scavenger receptors *Cd36*, *Stab1*, *Cd68*, *Cd14*, *Msr1*, *Cd44*, *Cxcl16*, *Clec7a*, *Scarb1*, *Olr1*, and *Cd163* between young and aged microglia (Fig. 1i, j). As CD36 was significantly downregulated in aged microglia and is a known receptor for the phagocytosis of oxidized phospholipids present in myelin debris [38], we focused on CD36. We found that a CD36 receptor antagonist (sulfo-*N*-succinimidyl oleate sodium, SSO) reduced phagocytosis by aged microglia in a concentration-dependent manner (Fig. 1k, l). Overexpression of CD36 through in vitro transcription resulted in a significant increase in phagocytosis in microglia isolated from young and aged mice together with microglia isolated from human subjects (Fig. 1m, n).

**Fig. 2:**
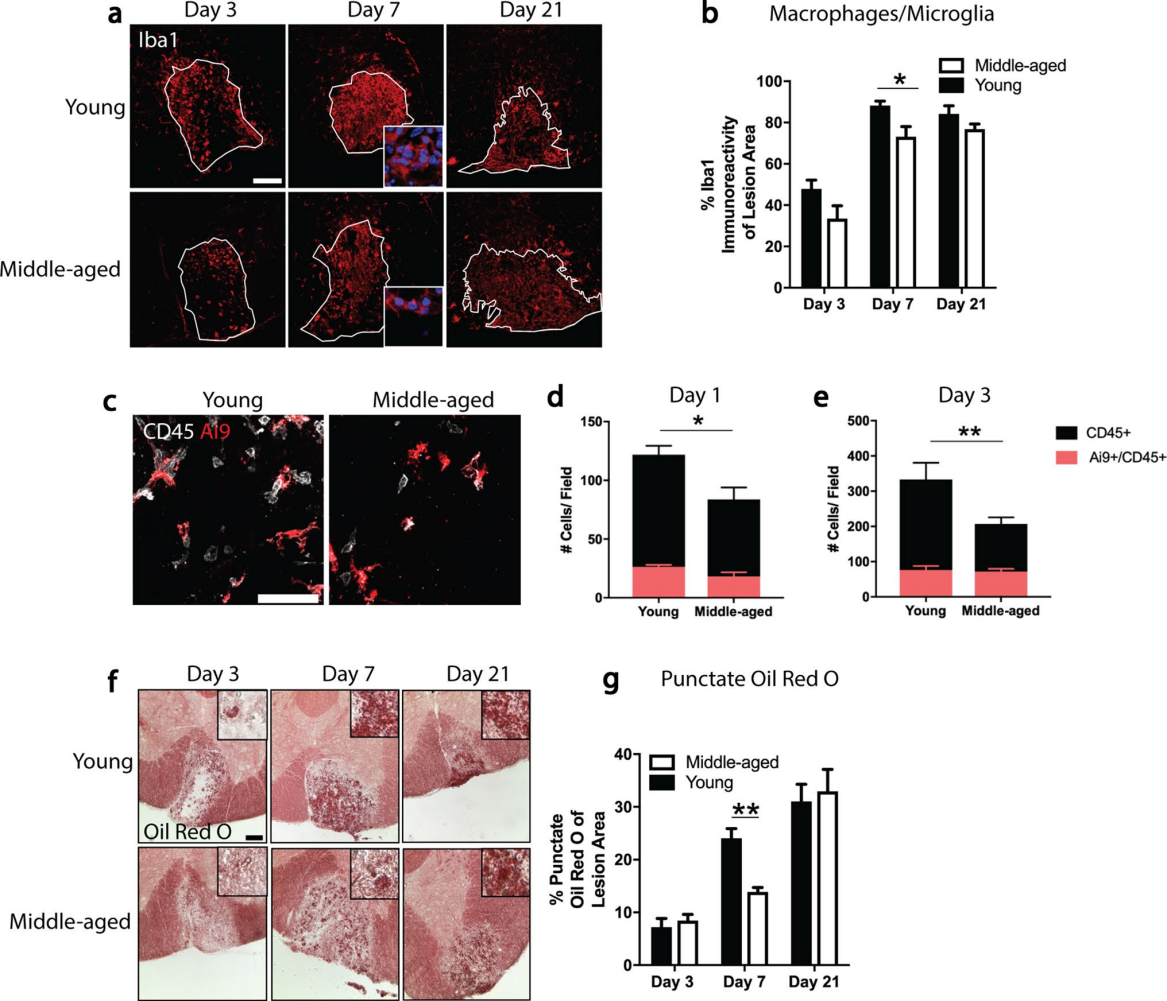
Middle-aged mice have decreased monocyte-derived macrophage recruitment and impaired phagocytosis. **a** Representative images of lesions from young (2–3 month) and middle-aged (9–12 month) mice stained with the macrophage/microglia marker-ionized calcium-binding adaptor molecule-1 (Iba1) at 3, 7, and 21 days post-demyelination. **b** Lesions in middle-aged mice have significantly less Iba1 immunoreactivity than lesions in young mice at 7 days. **c** Representative images of lysolecithin lesions from young and middle-aged CX3CR1^CreER^:Rosa26^TdT^ (Ai9) mice stained with an antibody against CD45. **d**, **e** The number of microglia (Ai9+/CD45+) within the lesion does not differ between young and middle-aged mice. The number of peripheral leukocytes (CD45+), however, is significantly less in middle-aged mice at both time points. **f** Representative bright field images of lesion epicenters from young and middle-aged mice stained with Oil Red O (ORO) at 3, 7, and 21 days post-demyelination. **g** Lesions in young mice have significantly more punctate ORO staining than lesions in middle-aged mice at 7 days post-demyelination. Values are represented as mean with the standard error of the mean. Results were analyzed with a two-way ANOVA with a Bonferroni’s post hoc test (**b**, **d**, **e**, **g**). For panels **b**, **d**, **e**, and **g**, between five and nine mice were analyzed per group. For panels **a** and **f**, scale bars equal 100 μm. For panel **c**, scale bars equal 50 μm. *p < 0.05; **p < 0.01

To assess whether these findings translated to an in vivo model, the demyelinating toxin lysolecithin was injected into the ventrolateral funiculus of young (2–3 months) and middle-aged (9–12 months) mice. This typically produces in young mice an initial complete demyelination followed by repopulation of OPCs by day 7 and remyelination by day 14–21 [19]. Analysis of lesion epicenter areas between young and middle-aged mice with the myelin stain, eriochrome cyanine, showed no statistically significant differences on days 3, 7, or 21 (Supplementary Fig. 1, online resource). Staining for the pan-macrophage/microglia marker Iba1 showed an initial low level of immunoreactivity in lesions of middle-aged mice (17% decrease compared to young mice) that eventually reached levels of young mice on day 21 (Fig. 2a, b). This delay was due to lower number of monocyte-derived macrophages rather than microglia in lesions of middle-aged animals when CX3CR1^CreER^:Rosa26^Tdt^ (Ai9) mice were used to discriminate Ai9+ microglia from Ai9− monocytes (24 h: 32% decrease; 72 h: 65% decrease) [36] (Fig. 2c–e). Phagocytosis in lesions of middle-aged mice was examined with Oil Red O (ORO) to detect neutral lipids [24, 41]; phagocytosed lipids concentrated in phagolysosomes have an intense, punctate appearance (Fig. 2f). Quantitation of punctate ORO showed significantly less ingested lipids on day 7 (42% decrease) in lesions of middle-age mice compared to lesions of young mice (Fig. 2g).

**Fig. 3:**
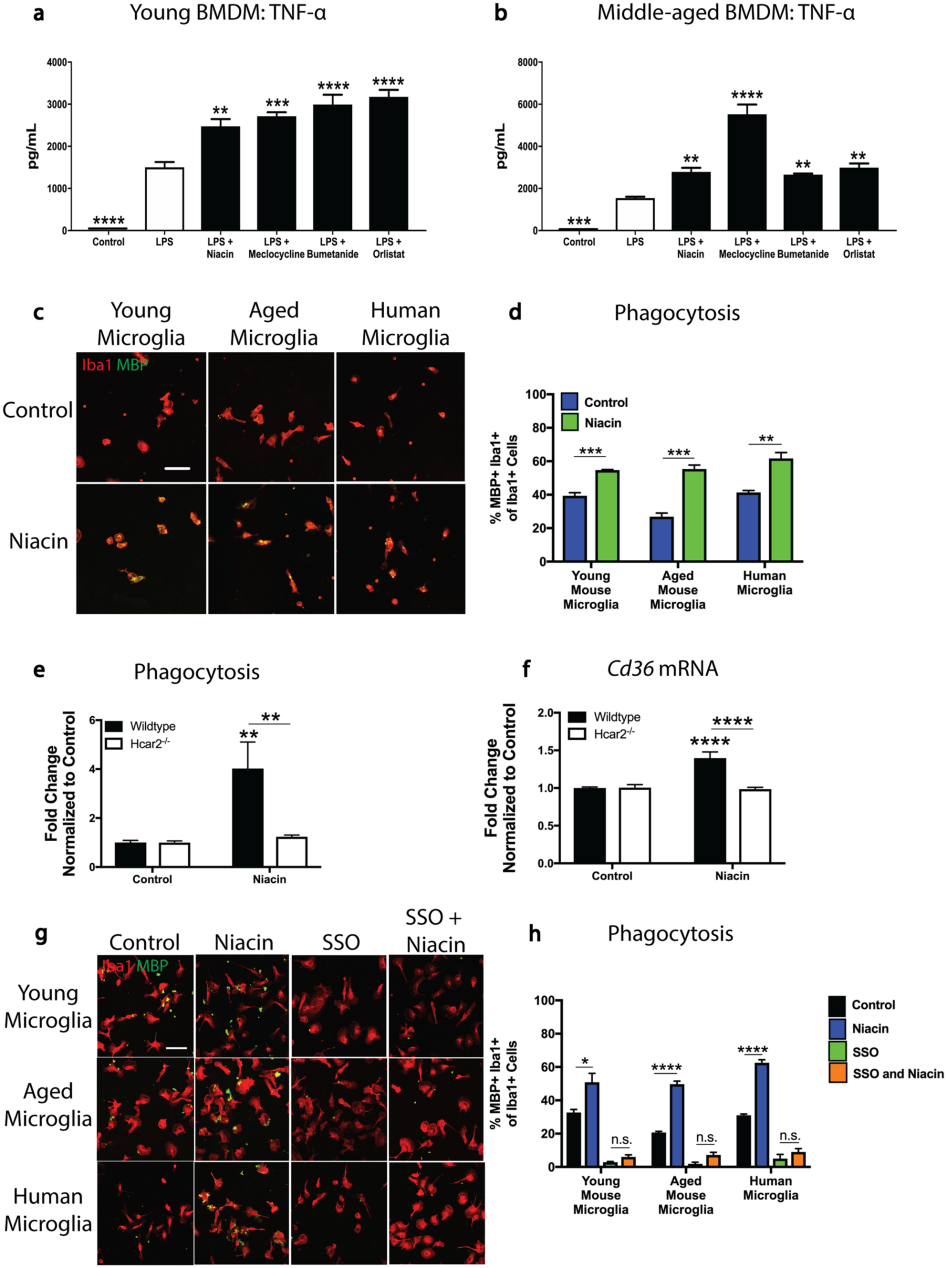
Niacin is a novel stimulator of phagocytosis and acts through the niacin receptor (Hcar2) to upregulate CD36. **a**, **b** ELISA for TNF-α shows medications that increase TNF-α production in LPS (100 ng/mL)-stimulated condition in young (**a**) and middle-aged (from 9 to 12-month-old animals) (**b**) mouse BMDM. Note that without LPS, the compounds did not elevate TNF-α (data not shown). **c** Representative images of young and aged (from > 15 month old) mouse microglia and human microglia exposed to myelin debris and treated with niacin (100 μM) and stained for Iba1 (red) and MBP (green). **d** Niacin (100 μM) by itself significantly increases the engulfment of MBP+ myelin debris within Iba1+ young and aged mouse microglia and human microglia. **e** Niacin (100 µM) by itself enhances phagocytosis and this is abrogated in Hcar2^*−/−*^ BMDM. **f** Niacin alone (100 µM) increases the expression of *Cd36* mRNA in wildtype but not Hcar2^−/−^ BMDM. **g** Representative images of young and aged mouse microglia and human microglia exposed to myelin debris and treated with control, niacin (100 μM), sulfo-*N*-succinimidyl oleate (SSO), and niacin together with SSO. These cells were stained for Iba1 (red) and MBP (green). **h** The niacin-mediated increase in phagocytosis is abrogated in young and aged mouse microglia as well as human microglia treated with a CD36 antagonist (SSO). For panels **a**, **b**, values are represented as mean with the standard error of the mean of quadruplicate cultures. For panel **d**, values are represented as mean with the standard error of the mean of triplicate cultures. An independent experiment was performed for each cell type. For panel **e**, values are represented as mean with the standard error of the mean pooled from two independent experiments of quadruplicate cultures each. For panel **f**, values are represented as mean with the standard error of the mean pooled from two independent experiments of triplicate cultures each. For panel **h**, values are represented as mean with the standard error of the mean of triplicate cultures. An independent experiment was performed for each cell type. For panels **a**, **b**, results were analyzed by one-way ANOVA with Dunnett post hoc test relative to the LPS group. For panel **d**, results were analyzed by one-tailed *t* test performed independently for each cell type. For panels **e**, **f**, results were normalized to the respective control mean value and then analyzed by two-way ANOVA with a Bonferroni’s post hoc test. For panel **h**, results were analyzed by one-way ANOVA with a Bonferroni’s post hoc test performed independently for each cell type. For panels **c**, **g**, scale bars equal 50 μm. *p < 0.05; **p < 0.01; ***p < 0.001; ****p < 0.0001

We previously screened a 1040 drug library for their capacity to enhance TNF-α production in lipopolysaccharide (LPS)-exposed adult human microglia and identified niacin (nicotinic acid, vitamin B3), meclocycline, bumetanide and orlistat with this effect [44]. Here, we used macrophages since they are a major phagocytic population in many neurological lesions [32], and can be purified easily from the bone marrow of young (2–3 months) and middle-aged (9–12 months) mice. All four compounds by themselves did not elicit an elevation of TNF-α (data not shown), but they significantly enhanced TNF-α levels in LPS-stimulated bone marrow-derived macrophages (BMDM) from both age groups (Fig. 3a, b). In considering which stimulator would be the best candidate for potential clinical use, we noted that meclocycline has an unfavorable safety profile, oral orlistat is not absorbed from the intestinal tract, and bumetanide has poor CNS penetrance (www.drugs.com). Niacin, however, is widely used and tolerated at high doses for prolonged periods and displays high bioavailability when taken orally to treat dyslipidemia [8, 25]. Moreover, the concentration we used of 100 μM is achievable in plasma in humans [6] and niacin is detected in the human brain after administration [16]. We assessed whether niacin is able to improve microglial phagocytosis of myelin debris and found a significant enhancement of myelin debris uptake by both young and aged mouse microglia, as well as human microglia (Fig. 3c, d).

Niacin exerts its anti-lipolytic effects through the niacin receptor, also known as GPR109A or the hydroxycarboxylic acid receptor 2 (Hcar2) [3, 25, 49]. To determine the involvement of this receptor, we isolated BMDM from wildtype and Hcar2^*−/−*^ mice. Multiplex Luminex analyses supported the TNF-α ELISA results and niacin induced elevation of several cytokines and chemokines in LPS-exposed wildtype BMDM that was abrogated in Hcar2^−/−^ cells (Supplementary Fig. 2a–h, online resource).

To determine whether niacin was binding to Hcar2 to mediate the increase in phagocytosis, we treated wildtype and Hcar2^−/−^ cells with niacin and only observed enhancement in phagocytosis in wildtype cells (Fig. 3e). To address potential scavenger receptors mediating this increase in phagocytosis, we assessed transcripts from wildtype and Hcar2^−/−^ BMDM treated with niacin. We found that *Cd68* and *Mertk* were not affected by niacin treatment (Supplementary Fig. 2i, j, online resource). However, niacin elevated *Cd36*, which is downregulated in aged microglia and important for the phagocytosis of myelin debris (Fig. 3f).

Next, we tested whether the niacin-mediated enhancement in phagocytosis was through CD36. Microglia were exposed to myelin and were treated with niacin in the presence of the CD36 receptor antagonist SSO. When niacin was added alone to microglia from young and aged mice, as well as from humans, a significant increase in phagocytosis was observed compared to control (Fig. 3g, h). In contrast, SSO ameliorated niacin-enhanced myelin phagocytosis (Fig. 3g, h). These results identify niacin as a novel stimulator of macrophage/microglia phagocytosis in culture that acts through Hcar2 to upregulate CD36 culminating in myelin engulfment.

**Fig. 4:**
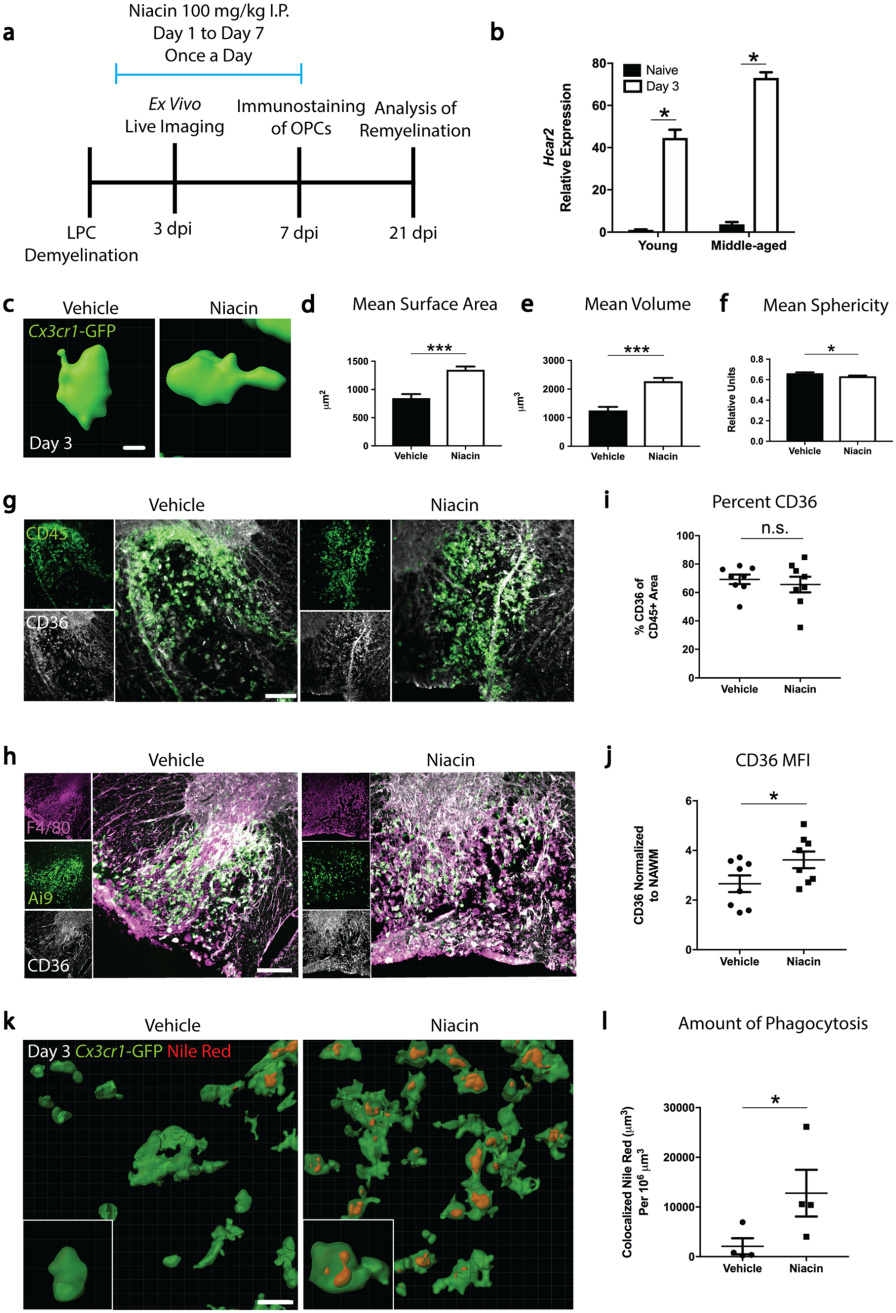
Niacin treatment of middle-aged mice alters macrophage/microglia morphology and promotes myelin phagocytosis. **a** Schematic depicting treatment paradigm with niacin. Middle-aged mice were injected with lysolecithin in either the dorsal or ventrolateral funiculi on day 0, after which niacin was administered intraperitoneally (I.P.) once a day at a dose of 100 mg/kg from day 1 to day 7. On day 3, lesioned dorsal spinal cord segments were isolated for ex vivo multiphoton live imaging. On day 7, ventrolateral lesions were processed for immunofluorescence staining for oligodendrocyte progenitor cells (OPCs). On day 21, remyelination was assessed by immunofluorescence staining and electron microscopy of ventrolateral lesions. **b** RNA transcript of the niacin receptor is upregulated in the lesion 3 days post-injury in both young and middle-aged mice. **c** Representative three-dimensional reconstructions of single CX3CR1^GFP/+^ macrophage/microglia in 3 day lesions from middle-aged mice administered with saline vehicle or niacin. **d**, **e** Niacin treatment significantly increases the mean surface area (**d**) and mean volume (**e**) of macrophages/microglia within the lesion. **f** Treatment with niacin significantly reduces the mean sphericity of macrophages/microglia within the lesion. **g** Representative images depicting lesions immunostained for CD45 (green) and CD36 (white) at 3 days post-demyelination from middle-aged demyelinated mice receiving either vehicle or niacin. **h** Representative images of lysolecithin lesions 7 days post-demyelination from middle-aged CX3CR1^CreER^:Rosa26^Tdt^ (Ai9) mice treated with vehicle or niacin once a day for seven days. Both F4/80+/Ai9+ microglia (both magenta and green) as well as F4/80+/Ai9− peripheral macrophages (only magenta) express CD36 (white). **i** Niacin treatment of middle-aged mice has no effect on the percentage of CD45+ cells expressing CD36 within lesions at 3 days post-demyelination; however, there was a significant increase in the mean fluorescence intensity (MFI) of CD36 on CD45+ cells within lesions (**g**, **j**). **k** Three-dimensional reconstruction of CX3CR1^GFP/+^ macrophages/microglia containing Nile Red-positive phagosomes in lesions from middle-aged mice, with images acquired through ex vivo multiphoton live imaging 3 days post-demyelination. **l** Niacin enhances the amount of phagocytosed Nile Red-labeled lipid debris. Values are represented as mean with the standard error of the mean. Results were analyzed with a Wald test and Benjamin-Hochberg *p* value adjustment (**b**), two-tail student’s *t* test **(d–f)** or a one-tail student’s *t* test **(i**, **j**, **l)**. For panel **b**, each data point was generated from the analysis of RNA pooled from 5 mice. For panels **d**, **e**, **f**, between 11 and 73 cells were quantified per mouse from 3 vehicle-treated mice and 4 niacin-treated mice. For panels **i**, **j**, **l**, each data point represents 1 mouse. For panels, **d**–**f**, **i**, **j**, **l**, *p < 0.05; **p < 0.01; ***p < 0.001; ****p < 0.0001; *n.s.* not significant. For panel **b**, *p < 0.0001. Scale bars equal 5 μm (**c**), 20 μm (**k**), and 100 μm (**g**, **h**)

Since *Hcar2* expression was elevated in demyelinating lesions (young: 4361% increase; middle-aged: 1896% increase; Fig. 4b), we asked whether Hcar2 is necessary for remyelination to proceed efficiently. We induced lysolecithin lesions in young wildtype and Hcar2^*−/−*^ mice and found no difference in the rate of OPC recruitment or remyelination, suggesting that Hcar2 is not necessary for spontaneous remyelination (Supplementary Fig. 3a, b, online resource). We next addressed whether pharmacological stimulation of Hcar2 with the systemic administration of niacin would be sufficient to enhance remyelination in middle-aged mice (Fig. 4a). Wildtype or *Cx3cr1*^GFP/+^:*Thy1*^YFP+^ mice, demyelinated with lysolecithin, were treated with saline vehicle or niacin (100 mg/kg IP) once a day, with treatment starting 24 h after lysolecithin (Fig. 4a). From the literature, this dose results in an initial spike in plasma concentration, after which the concentration is maintained at approximately 240 µM from 2 to 6 h post-administration [51]. From 6 to 9 h, the plasma concentration decreases to 120 µM, resulting in a concentration similar to the one achieved in humans taking 1 g of niacin [6]. In our experiments, live imaging revealed that the macrophages/microglia in niacin-treated middle-aged mice had higher mean surface area (59% increase) and volume (82% increase), and lower mean sphericity (that is, less ameboid; 4% decrease) (Fig. 4c–f), indicating that niacin enhanced their process extension and elevated their surveillance within lesions.

Since myelin debris is non-permissive for remyelination and needs to be removed for remyelination to occur [22, 37], we investigated the effect of niacin on myelin phagocytosis in vivo. Extending our tissue culture results (Fig. 3f), we found that niacin significantly increased the mean fluorescence intensity of CD36 expression by monocyte-derived macrophages and microglia within the lesion (Fig. 4g–j). To determine whether the elevated CD36 corresponded to lipid engulfment, we performed ex vivo live imaging experiments in which addition of the lipophilic dye, Nile Red, labels lipids that have been engulfed by *Cx3cr1*^GFP/+^ cells [39, 48]. We found that systemic niacin treatment significantly enhanced the amount of engulfed lipids within CX3CR1^GFP/+^ macrophages/microglia on day 3 in middle-aged mice (512% increase) (Fig. 4k, l). Niacin thus promotes the clearance of inhibitory myelin debris from lesions in middle-aged animals.

Other effects of niacin were examined. Ex vivo multiphoton live imaging of CX3CR1^GFP/+^ mice at day 3 showed that niacin did not influence the motility of macrophages/microglia within lesions in middle-aged animals (Supplementary Fig. 4, online resource). Niacin did not affect the inflammatory profile of circulating blood monocytes after 3 or 7 days of daily treatment (Supplementary Fig. 5, online resource). In lesions on both day 3 and day 7 (Supplementary Fig. 4b and 6a,b, online resource), niacin did not alter the density of CX3CR1^GFP/+^ or Iba1-stained macrophages/microglia, or changed the proportion of cells within the lesion that were microglia or infiltrating macrophages (Supplementary Fig. 6c,d, online resource). We conclude that niacin is not affecting circulating monocytes or the recruitment of macrophages/microglia into lesions in middle-aged animals, but that it promotes the intralesional phagocytic clearance of inhibitory myelin debris.

**Fig. 5:**
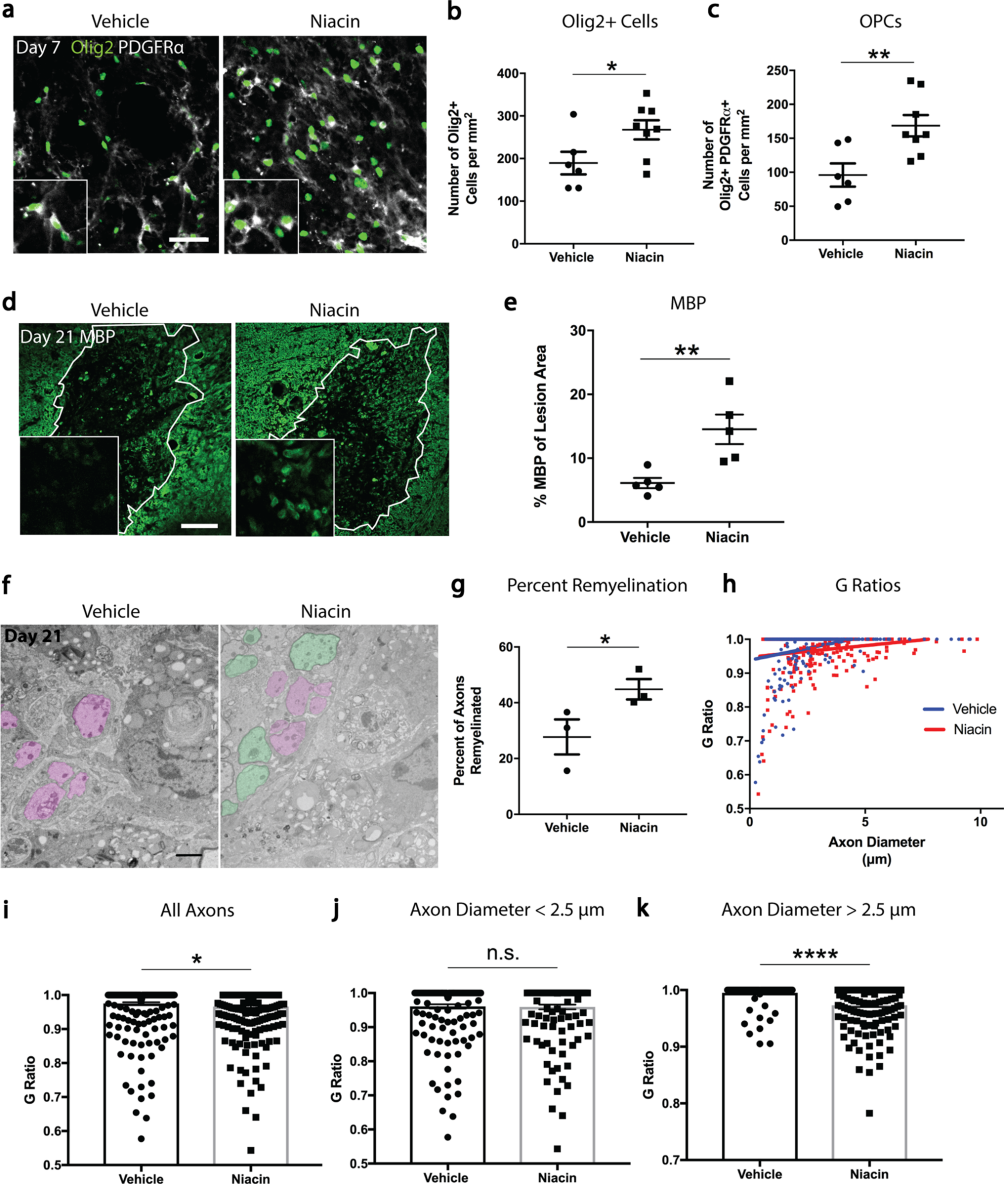
Niacin treatment of middle-aged mice enhances recruitment of OPCs and rejuvenates remyelination. **a** Representative images of lesion epicenters double-stained with Olig2 (green) and PDGFRα (white) from vehicle- and niacin-treated mice at 7 days post-demyelination. **b**, **c** Seven day lesions in niacin-treated mice have a significant increase in the densities of Olig2+ oligodendrocyte lineage cells and Olig2+PDGFRα+OPCs. **d** Representative images of lesion epicenters stained for MBP (green) from vehicle- and niacin-treated mice at 21 days post-demyelination. MBP-positive myelin rings (with zoomed images as inserts) are low in intensity whereas MBP-positive debris is high in intensity. **e** There is a significant increase in the percentage of MBP-positive area within the lesions of niacin-treated mice. **f** Representative electron micrographs of day 21 lesions from middle-aged mice treated with either vehicle or niacin. Demyelinated axons are depicted as magenta whereas remyelinated axons are shown in green**.g** Lesion cores from middle-aged mice treated with niacin have a significant increase in the percentage of axons that are remyelinated compared to lesions from vehicle-treated mice. **h** Graph displaying *g* ratios as a function of axon diameter from mice treated with either vehicle or niacin. **i** Lesions from middle-aged mice treated with niacin have a significant decrease in the mean *g* ratio (i.e. more remyelinated axons) across all axons analyzed. **j** Small caliber axons (diameter < 2.5 μm) do not differ in *g* ratio between vehicle and niacin treatment. **k** Lesions from middle-aged mice treated with niacin have significantly decreased mean *g* ratio of large caliber axons (diameter > 2.5 μm). Values are represented as mean with the standard error of the mean. For panels **b**, **c**, **e**, **g**, **i**, **j**, and **k**, results were analyzed with a one-tail student’s *t* test. For panels **b**, **c**, **e**, **g**, each data point represents 1 mouse. *p < 0.05; **p < 0.01; ****p < 0.0001; *n.s*. not significant. Scale bar equals 50 μm (**a**), 100 μm (**d**) and 2 μm (**f**)

With the removal of inhibitory myelin debris, daily niacin treatment increased the density of Olig2+ oligodendrocyte lineage cells (41% increase), including Olig2+PDGFRα+ OPCs (76% increase) (Fig. 5a–c) in demyelinated lesions in middle-aged mice. As niacin does not have a direct effect on OPCs in vitro (Supplementary Fig. 7, online resource), it seems likely that niacin is acting through the phagocytic removal of inhibitory myelin debris to enhance OPC recruitment within the lesion.

We examined whether systemic niacin treatment for the first 7 days after lysolecithin injection, when myelin breakdown and removal is largely occurring, was sufficient to promote remyelination on day 21 in middle-aged mice. This was first addressed by quantifying the percentage of MBP-positive rings within lesions, presumed to be newly formed myelin around axons. Niacin significantly increased the percentage of MBP-positive area within the lesion (138% increase) (Fig. 5d, e). To confirm that remyelination had occurred, we examined lesions by transmission electron microscopy (EM) (Fig. 5f). Lesions from mice treated with vehicle or niacin had the same density of axons (Supplementary Fig. 8, online resource), suggesting that niacin was not neurotoxic despite its potential for elevating cytokines in culture (Fig. 3); the safety was corroborated by a similar expression of the pro-inflammatory IL-1β in lesions between groups (Supplementary Fig. 9, online resource).

The EM images with quantitation throughout the lesion core showed that the percentage of remyelinated axons was elevated by niacin (62% increase) (Fig. 5g). *g* ratio measurements for myelin thickness (1 is complete demyelination) across all axonal calibers (Fig. 5h, i) provided an initial impression of lower *g* ratios (i.e., remyelinated) on larger axons in the niacin group (Fig. 5h), so we delineated small and large axons based on a 2.5 μm axonal caliber cutoff. The results show remyelination occurring similarly on small axons in both niacin and vehicle groups (Fig. 5j), but large axons were minimally remyelinated in middle-aged mice unless treated with niacin (Fig. 5k). These results demonstrate that systemic treatment with niacin improves remyelination in the aging CNS, specifically in larger caliber axons that otherwise would not be remyelinated in aging after lysolecithin-induced demyelination.

Recently, it was shown that the ability for macrophages/microglia to export phagocytosed cholesterol is important for preventing the formation of cholesterol crystals and lysosomal rupture within macrophages/microglia in demyelinated lesions, and that this is reduced in aging [5]. The cholesterol efflux is regulated by reverse cholesterol transporters such as adenosine triphosphate-binding cassette (ABC) A1 (ABCA1) and G1 (ABCG1) on the plasma membrane [31], which are in turn controlled by liver × receptor alpha [13] that was previously shown to enhance remyelination in the aging CNS [5]. We did not find that niacin elevated *Abca1* or *Abcg1* in our study (Supplementary Fig. 10, online resource), thereby suggesting for the future that the combination of niacin promoting myelin engulfment together with a drug to stimulate cholesterol efflux could be even more effective for remyelination in aging animals.

## Discussion

Several studies have found that the efficiency of remyelination is delayed in the aging CNS and that this deficiency is associated with an impaired macrophage/microglia response [14, 47, 53]. Macrophages/microglia are essential for remyelination as these cells phagocytose inhibitory myelin debris and provide growth factors for OPC maturation. With aging, macrophages/microglia display delayed lesion recruitment, decreased intralesional motility, as well as deficient phagocytosis, thereby contributing to a more inhibitory lesion microenvironment [5, 34, 39, 41, 53]. In this study, we confirmed these results using the lysolecithin model in young and middle-aged mice and examined whether remyelination could be enhanced by pharmacological stimulation of aging macrophages/microglia. We screened 1040 mostly generic medications from the NINDS Custom Collection II database for compounds that altered tumor necrosis factor-α secretion from activated human microglia [44]. From this screen, we discovered niacin, or vitamin B3, to be effective in stimulating tumor necrosis factor-α from human microglia. Furthermore, niacin was able to significantly enhance secretion of this cytokine from bone marrow-derived macrophages of young and older mice, in addition to enhancing phagocytosis in young and aged (from > 15 months donors) mouse microglia as well as human microglia through the scavenger receptor CD36.

As niacin has an extensive clinical history when used in dyslipidemia and is well tolerated, we examined whether systemic niacin treatment would be effective in improving the deficient remyelination observed in middle-aged mice. Indeed, daily treatment with niacin enhanced myelin debris clearance, promoted OPC recruitment, as well as enhanced remyelination in lesions. Altogether, these results identify a novel clinically approved medication that is able to significantly stimulate the innate immune system to enhance remyelination in the aging central nervous system.

Several approaches have been applied to accelerate remyelination in the young CNS [12]. These include directly enhancing oligodendrocyte differentiation and reducing the synthesis of inhibitory molecules such as chondroitin sulfate proteoglycans [9, 10, 20, 28, 33]. We have utilized the combination of amphotericin B and macrophage colony-stimulating factor to promote the activity of young macrophages [10]. These experiments have been conducted in young animals where the inflammatory response and OPC response are already very efficient. It is not clear whether these approaches will be successful in the CNS with increasing age due to the significant dysregulation in both the aging innate immune system as well as the OPC population. Furthermore, it is not entirely clear what results in remyelination failure in MS. Myelin debris within lesions is only one aspect, but there are likely several other mechanisms that contribute to the remyelination block in MS. As lysolecithin lesions in aging rodents do eventually completely remyelinate after 63 days, the lysolecithin model is not a very representative model of remyelination failure in MS [51]. The establishment and validation of better representative animal models of progressive MS will be instrumental in dissecting other pathological mechanisms underlying remyelination failure. In addition, as there is a strong correlation between increasing age with the onset of both primary and secondary progressive multiple sclerosis, it underscores the importance in establishing better animal models and discovering translational approaches to enhancing remyelination in the aging CNS [7, 15, 50].

Macrophages/microglia have been shown to have both beneficial and detrimental roles in remyelination [27]. As mentioned above, the beneficial roles include the clearance of inhibitory myelin debris as well as the provision of important growth factors and metabolites. Despite these benefits, these cells have also been shown to be detrimental through the secretion of free radicals and reactive oxygen species in addition to the release of potentially harmful pro-inflammatory cytokines [40]. As we found that niacin was able to significantly upregulate the secretion of several pro-inflammatory cytokines in vitro, including TNF-α which has been shown to be toxic in its soluble form through TNFR1 on oligodendrocytes [4], we were concerned that niacin treatment in middle-aged mice may exacerbate neurotoxicity. We do not think that this is the case, however, as we found no difference in axonal density with niacin treatment. Furthermore, niacin treatment of mice induced with experimental autoimmune encephalomyelitis, an inflammatory model of MS, improved disease score and minimized the degree of inflammatory infiltrates in the CNS [52]. As pro-inflammatory macrophages/microglia have been previously shown to be important for remyelination [1, 26, 30], we may be harnessing the beneficial aspects of these cells without causing significant cell death or neurotoxicity. It is also reassuring that we did not find elevation of pro-inflammatory monocytes in the circulation of niacin-treated mice, suggesting its safety in patients with inflammatory demyelinating diseases including MS. Altogether, these results further emphasize the potential clinical utility of using niacin for remyelination.

In conclusion, we provide a clinically relevant direction to reverse the deficient macrophage/microglia response in middle-aged mice that contributes to deficient remyelinating activity. We have identified niacin as a novel clinically approved medication that enhances macrophage/microglia phagocytosis of inhibitory debris*,* resulting in increased OPC recruitment and ultimately remyelination in the aging CNS. It is interesting to note that niacin treatment promoted the remyelination of large caliber axons, which would suggest that CNS regions that have predominantly small axons such as the corpus callosum may not be significantly affected by the pro-remyelinating activity of niacin. Alternately, our results suggest that small caliber axons are efficient at remyelinating spontaneously, but that large caliber axons require the intermediary of a stimulator such as niacin. Examining the effect of niacin in an inflammatory model of MS where newly generated oligodendrocytes can be fate mapped in different CNS regions will be an experimental approach to address whether this is indeed the case [29]. Finally, as phagocytosis is increasingly thought to be important in other conditions such as the clearance of Aβ in Alzheimer’s disease or α-synuclein in Parkinson’s disease, we would propose that niacin is a promising candidate for translation across many disorders where stimulating macrophages/microglia is a desired outcome.

## Electronic supplementary material

Below is the link to the electronic supplementary material.

**SupplementaryFigure1Therearenostatisticallysignificantdifferencesinlesionepicenterareabetweenyoungandmiddle-agemice.** Lesion epicenter areas were quantified using the myelin stain, eriochrome cyanine, at Days 3, 7, and 21 post-lysolecithin. No differences were detected between young and middle-age mice at any time point. Values are represented as mean with the standard error of the mean. Between 5 and 7 mice were analyzed per age group for each time point and results were analyzed with a 2-way ANOVA with a Bonferroni’s post hoc test. **SupplementaryFigure2Niacinisanovelstimulatorofyoungandmiddle-agedmacrophagesandactsthroughtheniacinreceptor,GPR109A(Hcar2).a–h.** The elevation of several cytokines/chemokines induced by niacin (100 μM) and LPS (100 ng/mL), beyond LPS alone, is lost in Hcar2^-/-^ BMDM. **i,j.** Niacin (100 μM) has no effect on the expression of *Cd68***(i)** or *Mertk***(j)** in both wildtype and Hcar2^-/-^ BMDM. For panels a – h, values are represented as mean with the standard error of the mean of quadruplicate cultures. For panels i – j, values are represented as mean with the standard error of the mean pooled from two independent experiments of triplicate cultures each. For panels a – h, results were analyzed by 1-way ANOVA with Dunnett post hoc test relative to the LPS group. For panels i – j, results were normalized to the respective control mean value and then analyzed by 2-way ANOVA with Bonferroni post hoc test. * p < 0.05; ** p < 0.01; *** p < 0.001 (a-h: compared to LPS; i-j: relative to wildtype). **SupplementaryFigure3ThereisnodifferenceinOPCrecruitmentorremyelinationbetweenwildtypeandHcar2**^**-/-**^**mice.a.** Lesions from wildtype and Hcar2^-/-^ mice do not have any difference in Olig2+ PDGFRα+ OPC recruitment 10 days post-demyelination. **b.** There is no difference in the area of MBP within the lesion between wildtype and Hcar2^-/-^ mice 10 days post-demyelination. Values are represented as mean with the standard error of the mean. Results were analyzed with a 2-tailed student’s *t* test (n.s. = not significant). Each data point represents 1 mouse. **SupplementaryFigure4Treatmentwithniacindoesnotenhancemacrophage/microgliamotility.a.** Representative diagram displaying displacement vectors (blue) of individual macrophages/microglia over *exvivo* multiphoton live imaging session in vehicle- and niacin-treated lesions 3 days post-demyelination. Thy1^YFP+^ axons are shown in white. **b.** Lesions from vehicle-treated and niacin-treated mice have no difference in the amount of *Cx3cr1*^GFP/+^ macrophages/microglia in the lesions at 3 days post-demyelination. **c,d.** Graphs showing no difference in the mean displacement **(c)** or track straightness **(d)** of individual macrophages/microglia in lesions from vehicle-treated and niacin-treated mice 3 days post-demyelination. Values are represented as mean with the standard error of the mean. Results were analyzed with a 2-tail student’s *t* test (n.s. = not significant). For panel b, each data point represents 1 mouse. For panels c and d, between 11 and 56 cells were quantified per mouse from 3 vehicle-treated mice and 3 niacin-treated mice. Scale bar equals 20 μm. **SupplementaryFigure5Treatmentwithniacindoesnotalterbloodmonocyteprofileafter3or7daysoftreatment.** Representative flow cytometry plots of blood monocytes isolated from demyelinated mice (**a**: Day 3; **c**: Day 7) treated with either saline vehicle or niacin 100 mg/kg IP once a day from Day 1 to Day 3 (**a**) or to Day 7 (**c**). There are no differences in the percentages of CD45+ CD11b+ CD115+ circulating monocytes, CD45+ CD11b+ CD115+ Ly6C^Hi^ pro-inflammatory monocytes, CD45+ CD11b+ CD115+ Ly6C^Int^ monocytes, or CD45+ CD11b+ CD115+ Ly6C^Lo^ patrolling monocytes between vehicle- and niacin-treated mice (**b**: Day 3; **d**: Day 7). In addition, there is no difference in the mean fluorescence intensity of Ly6C on circulating monocytes between vehicle- and niacin-treated mice. Values are represented as mean with the standard error of the mean. Results were analyzed with a 1-tail student’s *t* test (n.s. = not significant). Each data point represents 1 mouse. **SupplementaryFigure6Treatmentwithniacindoesnotaltermacrophage/microgliadensityafter3or7daysoftreatment.a.** Representative images depicting lesions immunostained for Iba1 at 3 and 7 days post-demyelination from middle-aged demyelinated mice receiving either saline vehicle or niacin once a day for 3 or 7 days at a dose of 100 mg/kg IP, with quantitation in **b*.*c.** Representative images of lysolecithin lesions from middle-aged CX3CR1^CreER^ :Rosa26^TdT^ Ai9 mice treated with either vehicle or niacin once a day for 7 days stained with an antibody against Iba1 (green). **d.** The number of microglia (Ai9+/Iba1+) within the lesion does not differ between vehicle- and niacin-treated mice on Day 7 post-lysolecithin. Values are represented as mean with the standard error of the mean. For panel b, between 6 and 8mice were analyzed per treatment group for each time point and results were analyzed with a 2-way ANOVA with a Bonferroni’s post hoc test. For panel d, results were analyzed with a 2-tail student’s *t* test (n.s. = not significant). Scale bars equal 100 μm. **SupplementaryFigure7TreatmentwithniacindoesnotaltertheprocessoutgrowthoradherenceofOPCsinculture.a.** Representative images of OPCs stained for the sulfatide O4 (green) and Hoechst (blue). **b,c.** Quantification of process outgrowth **(b)** and number of cells **(c)**, showing no difference between control- and niacin-treated OPCs. Values are represented as mean with the standard error of the mean. Results were analyzed with a 2-tail student’s *t* test (n.s. = not significant). Scale bar equals 200 μm. **SupplementaryFigure8Thereisnodifferenceinaxonaldensitybetweenlesionsfrommiddle-agedmicetreatedwitheithervehicleorniacin.** Quantification of axonal density from electron micrographs of lesion cores from 3 vehicle- and 3 niacin-treated mice at 21 days post-demyelination. Values are represented as mean with the standard error of the mean. Results were analyzed with a 1-tail student’s t test. Each data point represents 1 mouse (n.s. = not significant). **SupplementaryFigure9TreatmentwithniacindoesnotalterexpressionofIL-1βwithinlesionsfrommiddle-agedmice.a.** Representative images depicting lesions immunostained for CD45 (white) and IL-1β (red) at 3 days post-demyelination from middle-aged demyelinated mice receiving either saline vehicle or niacin once a day for 3 days at a dose of 100 mg/kg IP. **b.** There is no difference in the percentage of IL-1β associated with CD45+ cells in lesions from both groups. **c.** Normalized mean fluorescence intensity (MFI) of IL-1β between lesions from vehicle- and niacin-treated mice 3 days post-demyelination did not differ. Values are represented as mean with the standard error of the mean. Results were analyzed with a 1-tail student’s t-test and each data point represents 1 mouse (n.s. = not significant). Scale bar equals 100 μm. **SupplementaryFigure10Treatmentwithniacindoesnotalterexpressionofgenesinvolvedinreversecholesteroltransport.a,b,c.** Niacin alone (100 µM) has no effect on the expression of *Abca1***(a)**, *Abcg1***(b)**, and *Apoe***(c)** in both wildtype and Hcar*2*^*-/-*^ BMDM. Values are represented as mean with the standard error pooled from two independent experiments, of triplicate cultures each. Results were normalized to the respective control mean value and then analyzed by 2-way ANOVA with Bonferroni post hoc test (PDF 15875 kb)

## References

[1] Arnett HA, Mason J, Marino M, Suzuki K, Matsushima GK, Ting JP (2001). TNF alpha promotes proliferation of oligodendrocyte progenitors and remyelination. Nat Neurosci.

[2] Bellavance MA, Gosselin D, Yong VW, Stys PK, Rivest S (2015). Patrolling monocytes play a critical role in CX3CR1-mediated neuroprotection during excitotoxicity. Brain Struct Funct.

[3] Benyo Z, Gille A, Kero J, Csiky M, Suchankova MC, Nusing RM (2005). GPR109A (PUMA-G/HM74A) mediates nicotinic acid-induced flushing. J Clin Invest.

[4] Brambilla R, Ashbaugh JJ, Magliozzi R, Dellarole A, Karmally S, Szymkowski DE (2011). Inhibition of soluble tumour necrosis factor is therapeutic in experimental autoimmune encephalomyelitis and promotes axon preservation and remyelination. Brain.

[5] Cantuti-Castelvetri L, Fitzner D, Bosch-Queralt M, Weil MT, Su M, Sen P (2018). Defective cholesterol clearance limits remyelination in the aged central nervous system. Science.

[6] Carlson LA, Oro L, Ostman J (1968). Effect of a single dose of nicotinic acid on plasma lipids in patients with hyperlipoproteinemia. Acta Med Scand.

[7] Confavreux C, Vukusic S (2006). Natural history of multiple sclerosis: a unifying concept. Brain.

[8] Creider JC, Hegele RA, Joy TR (2012). Niacin: another look at an underutilized lipid-lowering medication. Nat Rev Endocrinol.

[9] Deshmukh VA, Tardif V, Lyssiotis CA, Green CC, Kerman B, Kim HJ (2013). A regenerative approach to the treatment of multiple sclerosis. Nature.

[10] Doring A, Sloka S, Lau L, Mishra M, van Minnen J, Zhang X (2015). Stimulation of monocytes, macrophages, and microglia by amphotericin B and macrophage colony-stimulating factor promotes remyelination. J Neurosci.

[11] Feng G, Mellor RH, Bernstein M, Keller-Peck C, Nguyen QT, Wallace M (2000). Imaging neuronal subsets in transgenic mice expressing multiple spectral variants of GFP. Neuron.

[12] Franklin RJM, Ffrench-Constant C (2017). Regenerating CNS myelin—from mechanisms to experimental medicines. Nat Rev Neurosci.

[13] Fu X, Menke JG, Chen Y, Zhou G, MacNaul KL, Wright SD (2001). 27-hydroxycholesterol is an endogenous ligand for liver X receptor in cholesterol-loaded cells. J Biol Chem.

[14] Gilson J, Blakemore WF (1993). Failure of remyelination in areas of demyelination produced in the spinal cord of old rats. Neuropathol Appl Neurobiol.

[15] Goldschmidt T, Antel J, Konig FB, Bruck W, Kuhlmann T (2009). Remyelination capacity of the MS brain decreases with disease chronicity. Neurology.

[16] Hankes LV, Coenen HH, Rota E, Langen KJ, Herzog H, Wutz W (1991). Effect of Huntington's and Alzheimer's diseases on the transport of nicotinic acid or nicotinamide across the human blood-brain barrier. Adv Exp Med Biol.

[17] Irvine KA, Blakemore WF (2008). Remyelination protects axons from demyelination-associated axon degeneration. Brain.

[18] Jung S, Aliberti J, Graemmel P, Sunshine MJ, Kreutzberg GW, Sher A (2000). Analysis of fractalkine receptor CX(3)CR1 function by targeted deletion and green fluorescent protein reporter gene insertion. Mol Cell Biol.

[19] Keough MB, Jensen SK, Yong VW (2015). Experimental demyelination and remyelination of murine spinal cord by focal injection of lysolecithin. J Vis Exp.

[20] Keough MB, Rogers JA, Zhang P, Jensen SK, Stephenson EL, Chen T (2016). An inhibitor of chondroitin sulfate proteoglycan synthesis promotes central nervous system remyelination. Nat Commun.

[21] Kiernan JA (1984). Chromoxane cyanine R. II. Staining of animal tissues by the dye and its iron complexes. J Microsc.

[22] Kotter MR, Li WW, Zhao C, Franklin RJ (2006). Myelin impairs CNS remyelination by inhibiting oligodendrocyte precursor cell differentiation. J Neurosci.

[23] Kotter MR, Setzu A, Sim FJ, Van Rooijen N, Franklin RJ (2001). Macrophage depletion impairs oligodendrocyte remyelination following lysolecithin-induced demyelination. Glia.

[24] Kotter MR, Zhao C, van Rooijen N, Franklin RJ (2005). Macrophage-depletion induced impairment of experimental CNS remyelination is associated with a reduced oligodendrocyte progenitor cell response and altered growth factor expression. Neurobiol Dis.

[25] Lukasova M, Malaval C, Gille A, Kero J, Offermanns S (2011). Nicotinic acid inhibits progression of atherosclerosis in mice through its receptor GPR109A expressed by immune cells. J Clin Invest.

[26] Mason JL, Suzuki K, Chaplin DD, Matsushima GK (2001). Interleukin-1beta promotes repair of the CNS. J Neurosci.

[27] McMurran CE, Jones CA, Fitzgerald DC, Franklin RJ (2016). CNS remyelination and the innate immune system. Front Cell Dev Biol.

[28] Mei F, Fancy SP, Shen YA, Niu J, Zhao C, Presley B (2014). Micropillar arrays as a high-throughput screening platform for therapeutics in multiple sclerosis. Nat Med.

[29] Mei F, Lehmann-Horn K, Shen YA, Rankin KA, Stebbins KJ, Lorrain DS (2016). Accelerated remyelination during inflammatory demyelination prevents axonal loss and improves functional recovery. Elife.

[30] Miron VE, Boyd A, Zhao JW, Yuen TJ, Ruckh JM, Shadrach JL (2013). M2 microglia and macrophages drive oligodendrocyte differentiation during CNS remyelination. Nat Neurosci.

[31] Moore KJ, Tabas I (2011). Macrophages in the pathogenesis of atherosclerosis. Cell.

[32] Mrdjen D, Pavlovic A, Hartmann FJ, Schreiner B, Utz SG, Leung BP (2018). High-dimensional single-cell mapping of central nervous system immune cells reveals distinct myeloid subsets in health, aging, and disease. Immunity.

[33] Najm FJ, Madhavan M, Zaremba A, Shick E, Karl RT, Factor DC (2015). Drug-based modulation of endogenous stem cells promotes functional remyelination in vivo. Nature.

[34] Natrajan MS, de la Fuente AG, Crawford AH, Linehan E, Nunez V, Johnson KR (2015). Retinoid X receptor activation reverses age-related deficiencies in myelin debris phagocytosis and remyelination. Brain.

[35] Neumann B, Baror R, Zhao C, Segel M, Dietmann S, Rawji KS (2019). Metformin restores CNS remyelination capacity by rejuvenating aged stem cells. Cell Stem Cell.

[36] Parkhurst CN, Yang G, Ninan I, Savas JN, Yates JR, Lafaille JJ (2013). Microglia promote learning-dependent synapse formation through brain-derived neurotrophic factor. Cell.

[37] Plemel JR, Manesh SB, Sparling JS, Tetzlaff W (2013). Myelin inhibits oligodendroglial maturation and regulates oligodendrocytic transcription factor expression. Glia.

[38] Podrez EA, Poliakov E, Shen Z, Zhang R, Deng Y, Sun M (2002). A novel family of atherogenic oxidized phospholipids promotes macrophage foam cell formation via the scavenger receptor CD36 and is enriched in atherosclerotic lesions. J Biol Chem.

[39] Rawji KS, Kappen J, Tang W, Teo W, Plemel JR, Stys PK (2018). Deficient surveillance and phagocytic activity of myeloid cells within demyelinated lesions in aging mice visualized by ex vivo live multiphoton imaging. J Neurosci.

[40] Rawji KS, Mishra MK, Yong VW (2016). Regenerative capacity of macrophages for remyelination. Front Cell Dev Biol.

[41] Ruckh JM, Zhao JW, Shadrach JL, van Wijngaarden P, Rao TN, Wagers AJ (2012). Rejuvenation of regeneration in the aging central nervous system. Cell Stem Cell.

[42] Saab AS, Nave KA (2017). Myelin dynamics: protecting and shaping neuronal functions. Curr Opin Neurobiol.

[43] Safaiyan S, Kannaiyan N, Snaidero N, Brioschi S, Biber K, Yona S (2016). Age-related myelin degradation burdens the clearance function of microglia during aging. Nat Neurosci.

[44] Samanani S, Mishra M, Silva C, Verhaeghe B, Wang J, Tong J (2013). Screening for inhibitors of microglia to reduce neuroinflammation. CNS Neurol Disord Drug Targets.

[45] Segel M, Neumann B, Hill MFE, Weber IP, Viscomi C, Zhao C (2019). Niche stiffness underlies the ageing of central nervous system progenitor cells. Nature.

[46] Shen S, Sandoval J, Swiss VA, Li J, Dupree J, Franklin RJ (2008). Age-dependent epigenetic control of differentiation inhibitors is critical for remyelination efficiency. Nat Neurosci.

[47] Shields SA, Gilson JM, Blakemore WF, Franklin RJ (1999). Remyelination occurs as extensively but more slowly in old rats compared to young rats following gliotoxin-induced CNS demyelination. Glia.

[48] Stirling DP, Cummins K, Mishra M, Teo W, Yong VW, Stys P (2014). Toll-like receptor 2-mediated alternative activation of microglia is protective after spinal cord injury. Brain.

[49] Tunaru S, Kero J, Schaub A, Wufka C, Blaukat A, Pfeffer K (2003). PUMA-G and HM74 are receptors for nicotinic acid and mediate its anti-lipolytic effect. Nat Med.

[50] Tutuncu M, Tang J, Zeid NA, Kale N, Crusan DJ, Atkinson EJ (2013). Onset of progressive phase is an age-dependent clinical milestone in multiple sclerosis. Mult Scler.

[51] Yang R, He J, Wang Y (2016). Activation of the niacin receptor HCA2 reduces demyelination and neurofilament loss, and promotes functional recovery after spinal cord injury in mice. Eur J Pharmacol.

[52] Zhang J, Chen J, Li Y, Cui X, Zheng X, Roberts C (2008). Niaspan treatment improves neurological functional recovery in experimental autoimmune encephalomyelitis mice. Neurobiol Dis.

[53] Zhao C, Li WW, Franklin RJ (2006). Differences in the early inflammatory responses to toxin-induced demyelination are associated with the age-related decline in CNS remyelination. Neurobiol Aging.

