# Correction to: Niacin-mediated rejuvenation of macrophage/microglia enhances remyelination of the aging central nervous system

**DOI:** 10.1007/s00401-020-02146-6

**Published:** 2020-03-24

**Authors:** Khalil S. Rawji, Adam M. H. Young, Tanay Ghosh, Nathan J. Michaels, Reza Mirzaei, Janson Kappen, Kathleen L. Kolehmainen, Nima Alaeiilkhchi, Brian Lozinski, Manoj K. Mishra, Annie Pu, Weiwen Tang, Salma Zein, Deepak K. Kaushik, Michael B. Keough, Jason R. Plemel, Fiona Calvert, Andrew J. Knights, Daniel J. Gaffney, Wolfram Tetzlaff, Robin J. M. Franklin, V. Wee Yong

**Affiliations:** 1 Department of Clinical Neurosciences, Hotchkiss Brain Institute, University of Calgary, 3330 Hospital Drive, Calgary, AB T2N 4N1 Canada; 2 Wellcome-MRC Cambridge Stem Cell Institute, University of Cambridge, Cambridge, UK; 3 University of British Columbia, ICORD, Vancouver, Canada; 4 University of Alberta, Edmonton, Canada; 5 Wellcome Trust Sanger Institute, Hinxton, Cambridge, UK

## Correction to: Acta Neuropathologica 10.1007/s00401-020-02129-7

The article Niacin‑mediated rejuvenation of macrophage/microglia enhances remyelination of the aging central nervous system, written by Khalil S. Rawji, Adam M.H. Young, Tanay Ghosh, Nathan J. Michaels, Reza Mirzaei, Janson Kappen, Kathleen L. Kolehmainen, Nima Alaeiilkhchi, Brian Lozinski, Manoj K. Mishra, Annie Pu, Weiwen Tang, Salma Zein, Deepak K. Kaushik, Michael B. Keough, Jason R. Plemel, Fiona Calvert, Andrew J. Knights, Daniel J. Gaffney, Wolfram Tetzlaff, Robin J. M. Franklin and V. Wee Yong, was originally published electronically on the publisher’s internet portal on 6th February 2020 without open access.With the author(s)’ decision to opt for Open Choice the copyright of the article changed on 26th February 2020 to © The Author(s) 2020 and the article is forthwith distributed under a Creative Commons Attribution 4.0 International License (https://creativecommons.org/licenses/by/4.0/), which permits use, sharing, adaptation, distribution and reproduction in any medium or format, as long as you give appropriate credit to the original author(s) and the source, provide a link to the Creative Commons licence, and indicate if changes were made.

The original article has been corrected.

