# Supplementary material for: Niacin-mediated rejuvenation of macrophage/microglia enhances remyelination of the aging central nervous system
Source: Acta Neuropathol. 2020 Feb 6;139(5):893–909. doi: 10.1007/s00401-020-02129-7 (PMC7181452; doi:10.1007/s00401-020-02129-7)

Supplementary fig. 1, online resource

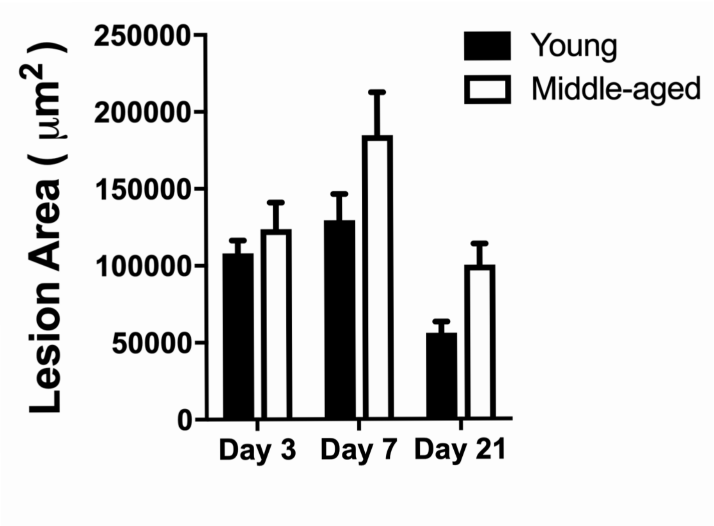

Supplementary fig. 2, online resource

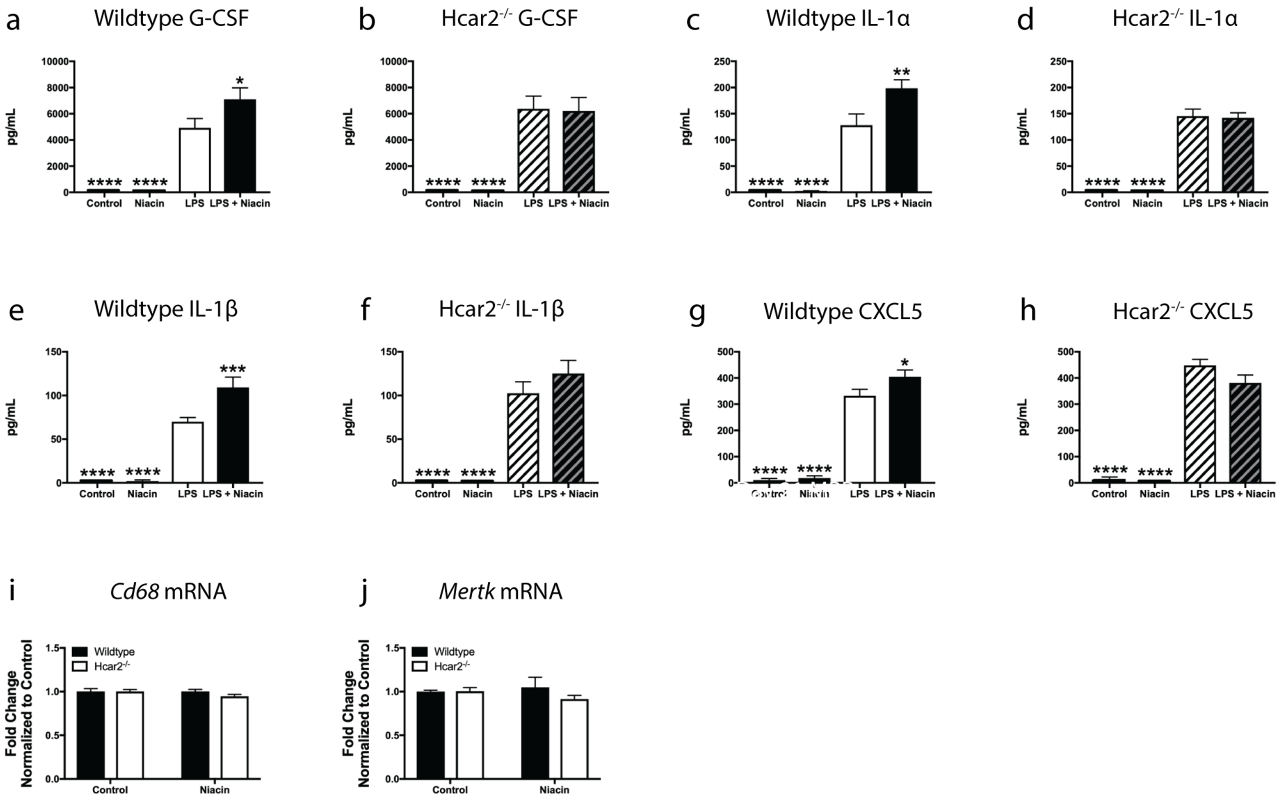

Supplementary fig. 3, online resource

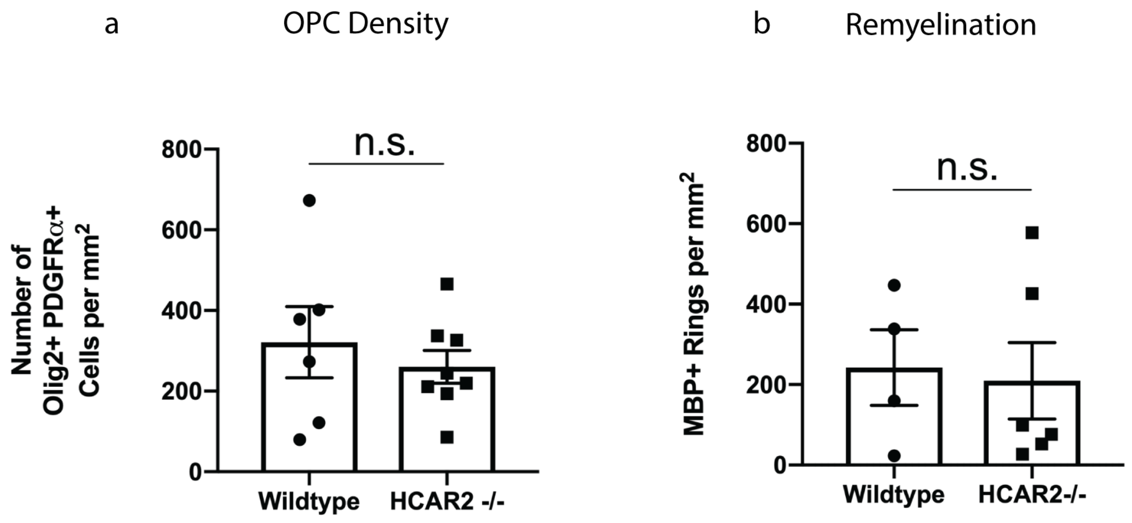

Supplementary fig. 4, online resource

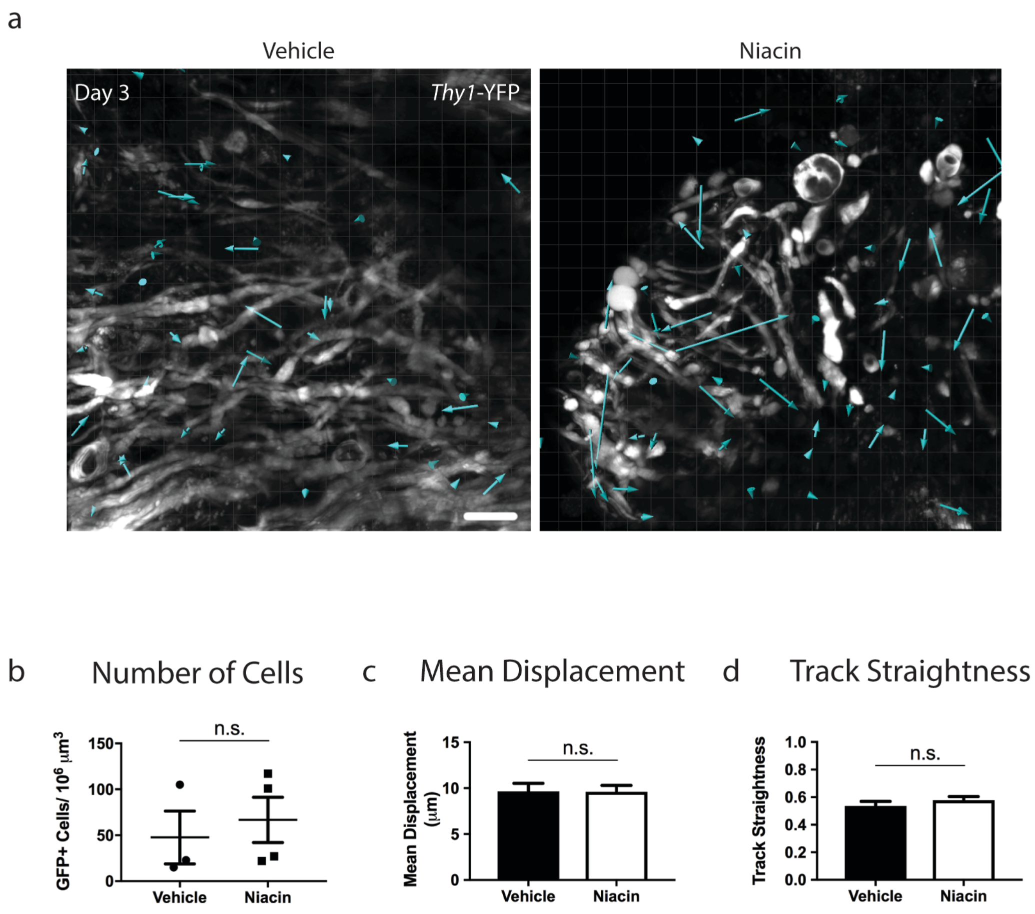

Supplementary fig. 5, online resource

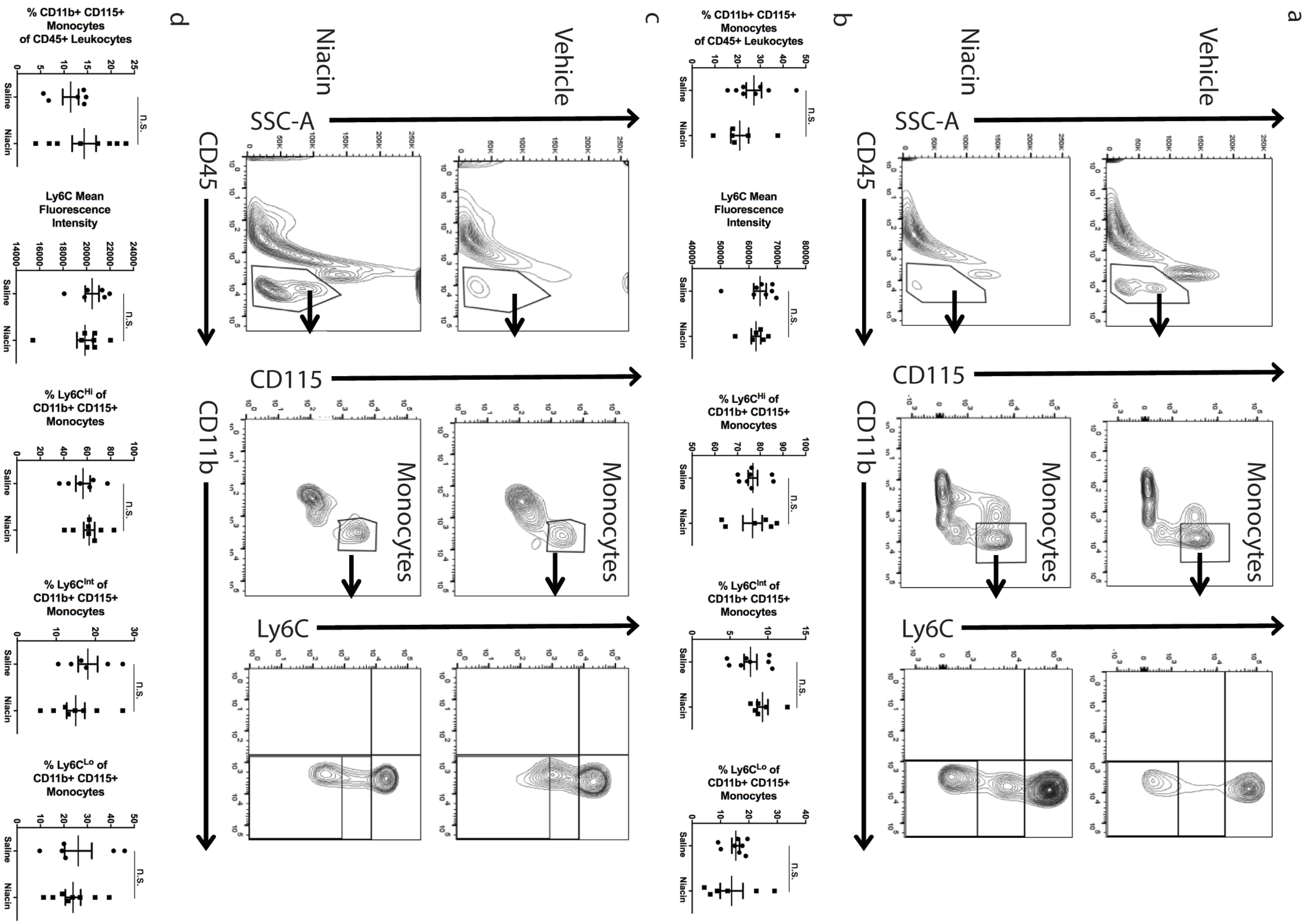

Supplementary fig. 6, online resource

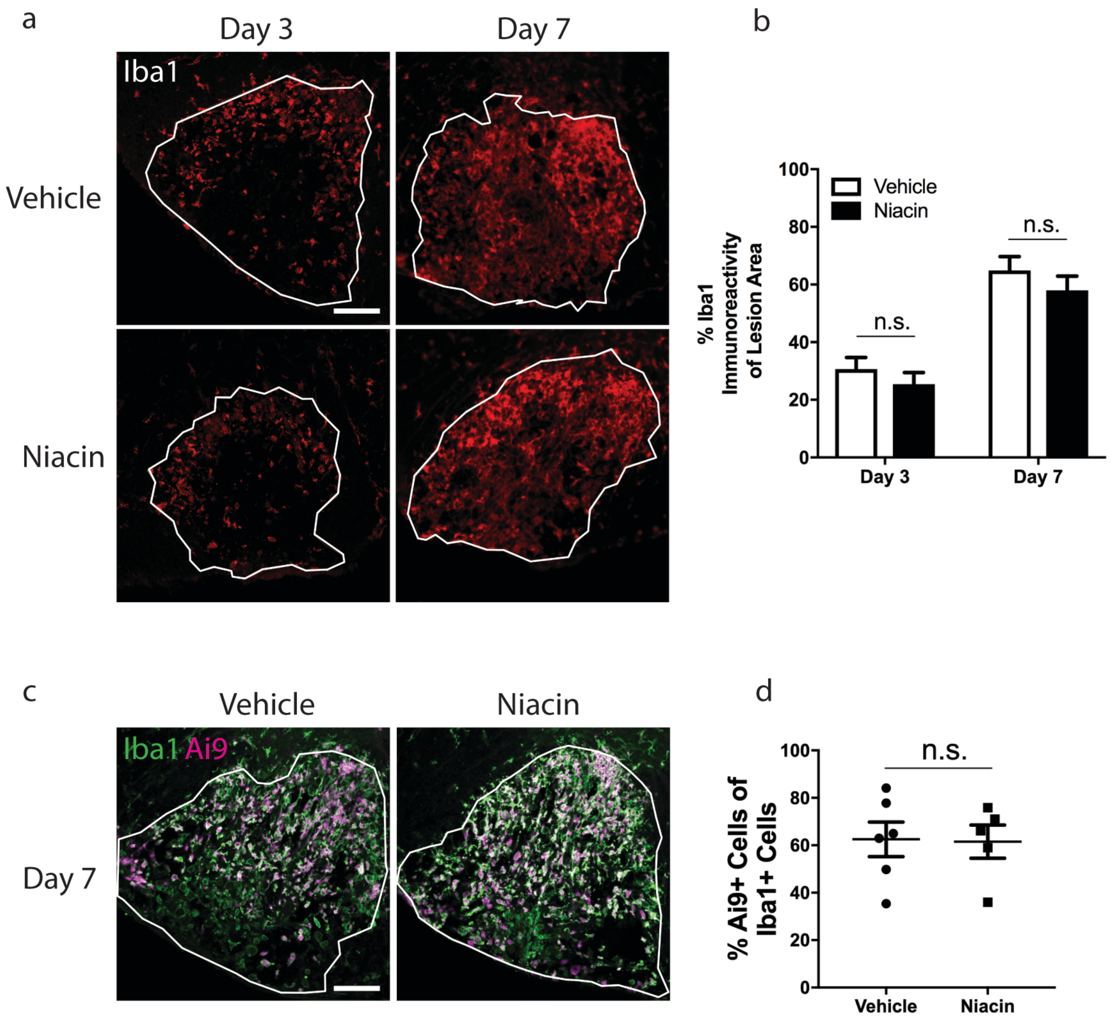

Supplementary fig. 7, online resource

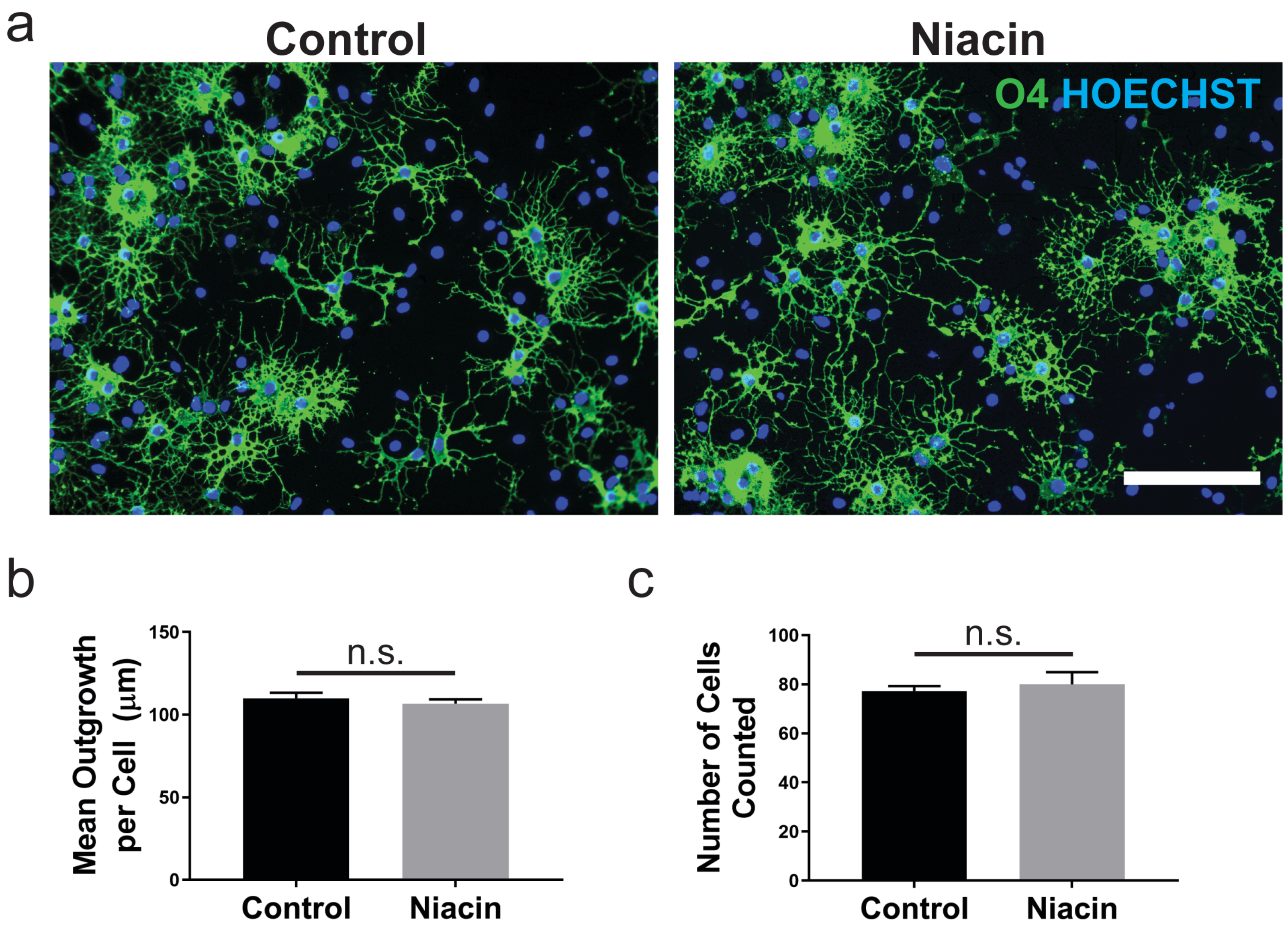

Supplementary fig. 8, online resource

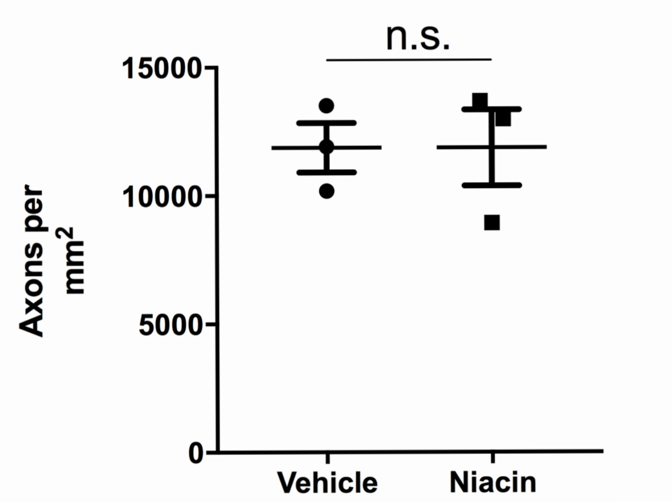

Supplementary fig. 9, online resource

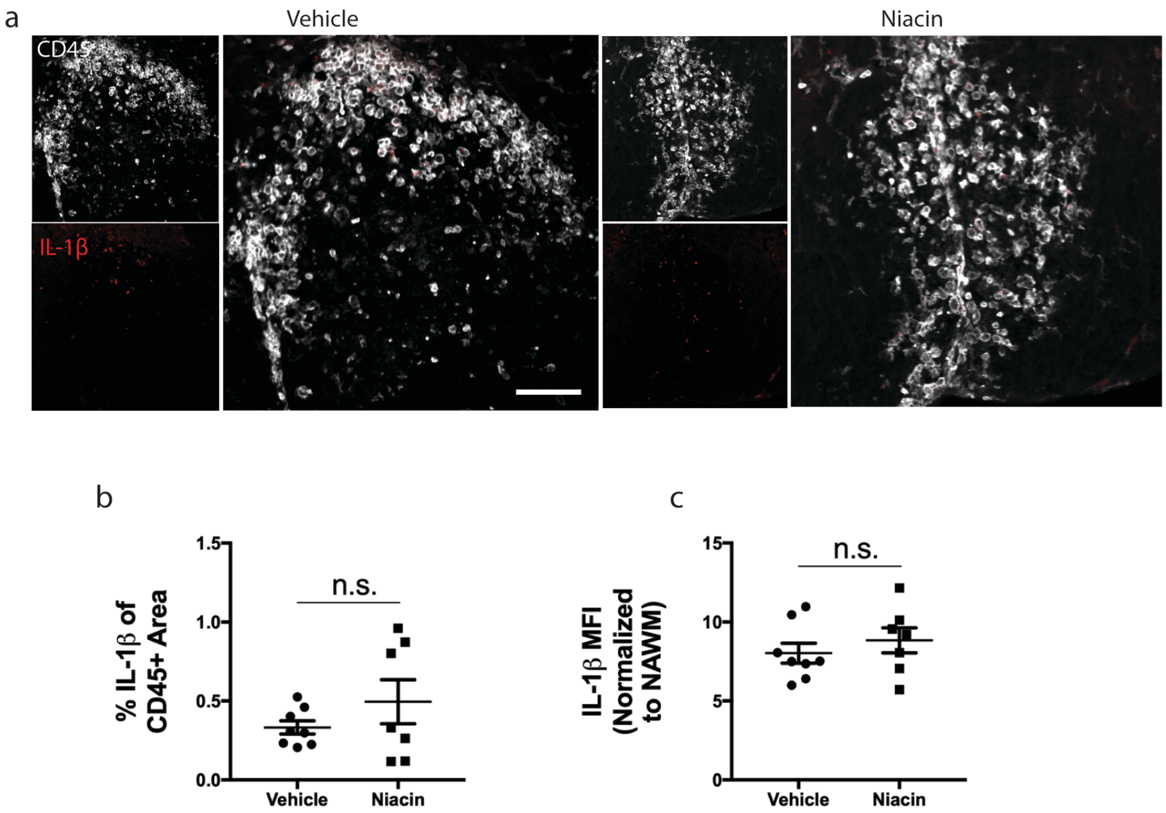

Supplementary fig. 10, online resource

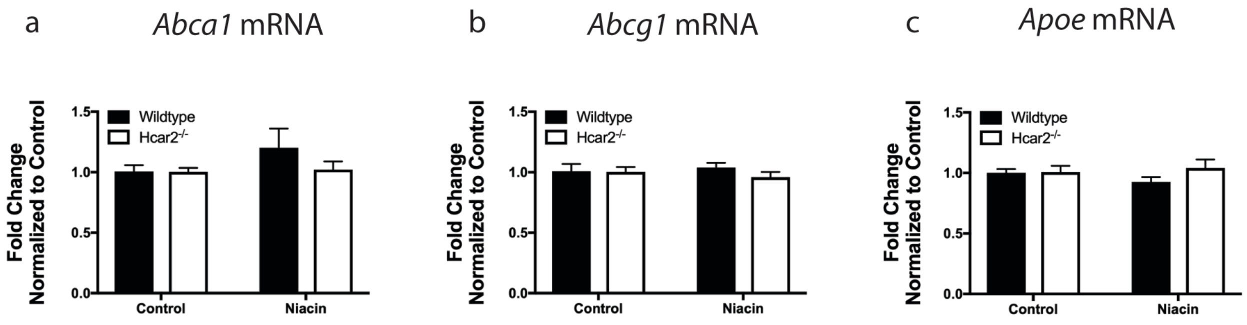

Supplement: Supplementary file 1 — SupplementaryFigure1Therearenostatisticallysignificantdifferencesinlesionepicenterareabetweenyoungandmiddle-agemice. Lesion epicenter areas were quantified using the myelin stain, eriochrome cyanine, at Days 3, 7, and 21 post-lysolecithin. No differences were detected between young and middle-age mice at any time point. Values are represented as mean with the standard error of the mean. Between 5 and 7 mice were analyzed per age group for each time point and results were analyzed with a 2-way ANOVA with a Bonferroni’s post hoc test. SupplementaryFigure2Niacinisanovelstimulatorofyoungandmiddle-agedmacrophagesandactsthroughtheniacinreceptor,GPR109A(Hcar2).a–h. The elevation of several cytokines/chemokines induced by niacin (100 μM) and LPS (100 ng/mL), beyond LPS alone, is lost in Hcar2-/- BMDM. i,j. Niacin (100 μM) has no effect on the expression of Cd68(i) or Mertk(j) in both wildtype and Hcar2-/- BMDM. For panels a – h, values are represented as mean with the standard error of the mean of quadruplicate cultures. For panels i – j, values are represented as mean with the standard error of the mean pooled from two independent experiments of triplicate cultures each. For panels a – h, results were analyzed by 1-way ANOVA with Dunnett post hoc test relative to the LPS group. For panels i – j, results were normalized to the respective control mean value and then analyzed by 2-way ANOVA with Bonferroni post hoc test. * p < 0.05; ** p < 0.01; *** p < 0.001 (a-h: compared to LPS; i-j: relative to wildtype). SupplementaryFigure3ThereisnodifferenceinOPCrecruitmentorremyelinationbetweenwildtypeandHcar2-/-mice.a. Lesions from wildtype and Hcar2-/- mice do not have any difference in Olig2+ PDGFRα+ OPC recruitment 10 days post-demyelination. b. There is no difference in the area of MBP within the lesion between wildtype and Hcar2-/- mice 10 days post-demyelination. Values are represented as mean with the standard error of the mean. Results were analyzed with a 2-tailed student’s [file 401_2020_2129_MOESM1_ESM.pdf]
